# Spatially restricted drivers and transitional cell populations cooperate with the microenvironment in untreated and chemo-resistant pancreatic cancer

**DOI:** 10.1038/s41588-022-01157-1

**Published:** 2022-08-22

**Authors:** Daniel Cui Zhou, Reyka G. Jayasinghe, Siqi Chen, John M. Herndon, Michael D. Iglesia, Pooja Navale, Michael C. Wendl, Wagma Caravan, Kazuhito Sato, Erik Storrs, Chia-Kuei Mo, Jingxian Liu, Austin N. Southard-Smith, Yige Wu, Nataly Naser Al Deen, John M. Baer, Robert S. Fulton, Matthew A. Wyczalkowski, Ruiyang Liu, Catrina C. Fronick, Lucinda A. Fulton, Andrew Shinkle, Lisa Thammavong, Houxiang Zhu, Hua Sun, Liang-Bo Wang, Yize Li, Chong Zuo, Joshua F. McMichael, Sherri R. Davies, Elizabeth L. Appelbaum, Keenan J. Robbins, Sara E. Chasnoff, Xiaolu Yang, Ashley N. Reeb, Clara Oh, Mamatha Serasanambati, Preet Lal, Rajees Varghese, Jay R. Mashl, Jennifer Ponce, Nadezhda V. Terekhanova, Lijun Yao, Fang Wang, Lijun Chen, Michael Schnaubelt, Rita Jui-Hsien Lu, Julie K. Schwarz, Sidharth V. Puram, Albert H. Kim, Sheng-Kwei Song, Kooresh I. Shoghi, Ken S. Lau, Tao Ju, Ken Chen, Deyali Chatterjee, William G. Hawkins, Hui Zhang, Samuel Achilefu, Milan G. Chheda, Stephen T. Oh, William E. Gillanders, Feng Chen, David G. DeNardo, Ryan C. Fields, Li Ding

**Affiliations:** 1 Department of Medicine, Washington University in St Louis, St Louis, MO USA; 2 McDonnell Genome Institute, Washington University in St Louis, St Louis, MO USA; 3 Department of Surgery, Washington University in St Louis, St Louis, MO USA; 4 Siteman Cancer Center, Washington University in St Louis, St Louis, MO USA; 5 Department of Pathology and Immunology, Washington University in St Louis, St Louis, MO USA; 6 Department of Genetics, Washington University in St Louis, St Louis, MO USA; 7 Department of Mathematics, Washington University in St Louis, St Louis, MO USA; 8 Department of Otolaryngology–Head & Neck Surgery, Washington University in St Louis, St Louis, MO USA; 9 Department of Bioinformatics and Computational Biology, University of Texas MD Anderson Cancer Center, Houston, TX USA; 10 Department of Pathology, Johns Hopkins University School of Medicine, Baltimore, MD USA; 11 Department of Radiation Oncology, Washington University in St Louis, St Louis, MO USA; 12 Department of Cell Biology and Physiology, Washington University in St Louis, St Louis, MO USA; 13 Department of Neurological Surgery, Washington University in St Louis, St Louis, MO USA; 14 Department of Radiology, Washington University in St Louis, St Louis, MO USA; 15 Department of Cell and Developmental Biology and Epithelial Biology Center, Vanderbilt University School of Medicine, Vanderbilt, TN USA; 16 Department of Computer Science and Engineering, Washington University in St Louis, St Louis, MO USA; 17 Department of Pathology, University of Texas MD Anderson Cancer Center, Houston, TX USA

**Keywords:** Pancreatic cancer, Cancer microenvironment

## Abstract

Pancreatic ductal adenocarcinoma is a lethal disease with limited treatment options and poor survival. We studied 83 spatial samples from 31 patients (11 treatment-naïve and 20 treated) using single-cell/nucleus RNA sequencing, bulk-proteogenomics, spatial transcriptomics and cellular imaging. Subpopulations of tumor cells exhibited signatures of proliferation, KRAS signaling, cell stress and epithelial-to-mesenchymal transition. Mapping mutations and copy number events distinguished tumor populations from normal and transitional cells, including acinar-to-ductal metaplasia and pancreatic intraepithelial neoplasia. Pathology-assisted deconvolution of spatial transcriptomic data identified tumor and transitional subpopulations with distinct histological features. We showed coordinated expression of TIGIT in exhausted and regulatory T cells and Nectin in tumor cells. Chemo-resistant samples contain a threefold enrichment of inflammatory cancer-associated fibroblasts that upregulate metallothioneins. Our study reveals a deeper understanding of the intricate substructure of pancreatic ductal adenocarcinoma tumors that could help improve therapy for patients with this disease.

## Abstract (web summary)

A multi-omic analysis of pancreatic cancer identifies spatially resolved, heterogeneous cell populations including transitional cell types. Analysis of primary samples identifies treatment-related changes in cross-talk between tumor and stromal cells.

## Main

Pancreatic ductal adenocarcinoma (PDAC) has an 11% 5-yr survival rate^1^ due to late detection, early metastases and therapy resistance^2–6^. First-line treatment is surgery followed by radiation and/or chemotherapy^7,8^, with immunotherapy options being limited^9,10^. Drivers such as *KRAS*, *TP53*, *CDKN2A* and *SMAD4* (HUGO Gene Nomenclature Committee at the European Bioinformatics Institute, https://www.genenames.org/) have been identified^11^ as have transcriptional subtypes of classical and basal-like^12,13^.

Single-cell technologies enable analysis regardless of tumor content and facilitate dissection of the tumor microenvironment (TME), whose role in PDAC remains largely unknown. For instance, cancer-associated fibroblast (CAF) subtypes have been identified and cytotoxic natural killer (NK) and CD8^+^ T cells are often numerically and functionally impaired^14–17^. This creates an immunosuppressed, pro-tumorigenic environment, but how this occurs is poorly understood^18,19^. There is a growing appreciation surrounding acinar-to-ductal metaplasia (ADM), in which acinar cells start expressing ductal markers. Animal models posit acinar cells as the origin of PDAC when KRAS(G12D) is expressed^20–23^, but this hypothesis is difficult to evaluate in humans due to the paucity of acinar and ADM cells sampled at single-cell resolution^24–28^. Recent efforts have focused on acinar heterogeneity in chronic pancreatitis^29^ and healthy human pancreas^30^, but adequate sampling of ADM cells is still lacking.

As part of the Human Tumor Atlas Network consortium, we used a spatially distinct, multi-sampling approach to analyze 83 PDAC samples across 31 patients^31^. Samples are physically separate from one another, which allowed interrogating both inter- and intra-tumor heterogeneity via extensive omics, including bulk DNA and RNA sequencing (RNA-seq), bulk proteomics and phosphoproteomics, single-cell and single-nucleus RNA-seq (scRNA-seq and snRNA-seq, respectively), cellular imaging and spatial transcriptomics. We identified and validated transitional populations and their associated molecular signatures along the spectrum from normal pancreas to PDAC that were previously proposed in mouse models. We characterized differential impact of chemotherapy on the abundance and transcriptional programs of tumor and stroma populations using multi-omic approaches. We highlight the necessity of spatial sequencing for polyclonal/heterogeneous PDAC tumor characterization.

## Results

### Study design and overview of the study cohort

**Fig. 1:**
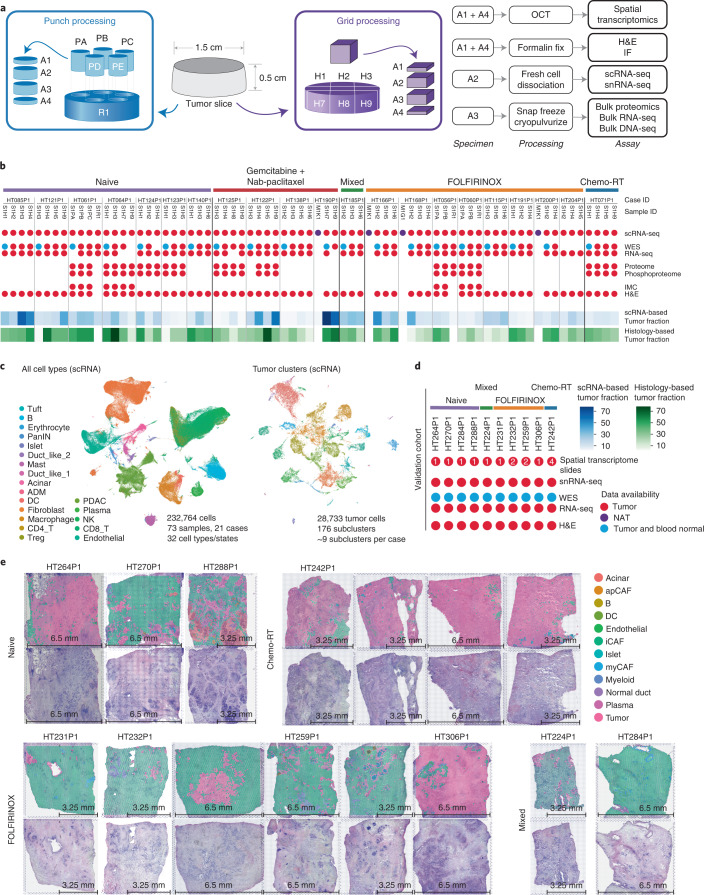
Sampling strategy and cohort overview. **a**, Spatial sampling approach. At least two punches or grids were selected from each tumor for comprehensive imaging and omics characterization. Prefixes: ‘P’ denotes tissue punches, ‘H’ denotes tissue grids, ‘R’ denotes remainder tissue and ‘A’ denotes a piece of tissue. Each associated piece of tissue was processed in a systematic fashion and utilized for the listed assays. **b**, Top, data overview of the cohort. Samples are organized by treatment status. M1K1 and M1G1 denote normal adjacent tissue (NAT) samples. Dots for each associated assay indicate data availability: red for tumor tissue, purple for NAT, and blue for tumor tissue and blood normal. Bottom, scRNA-based (blue) and histology-based estimates (green) of tumor purity (Methods). **c**, Overview of all cell types profiled in the scRNA-seq cohort. The left UMAP shows that a total of 232,764 cells were profiled from 73 samples and 21 cases, representing 32 cell types or states (colored by cell types). The right UMAP shows the 28,733 tumor cells subset (colored by sample). **d**, Data overview of the validation cohort. Treatment status is listed on the top followed by sample name and data availability. Dots for each associated assay indicate data availability: red for tumor tissue, and blue for tumor tissue and blood normal. For spatial transcriptome slides, the number indicated in the circle denotes the number of spatial transcriptome slides generated from that sample. **e**, Overview of spatial transcriptomics cohort. Samples are organized according to treatment groups. For each sample, the bottom image shows the H&E and the top image has cell types overlayed. DC, Dendritic Cells; ID, Identification; IF, Immunofluoresence; IMC, Immunohistochemistry.

**Extended Data Fig. 1:**
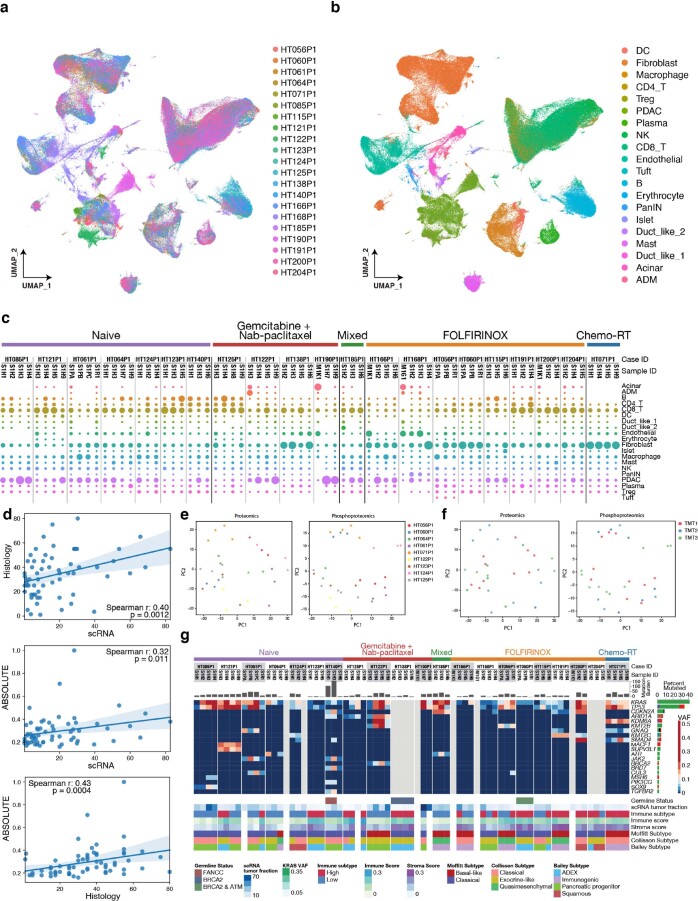
Details of Data Cohort Overview. **a)** All 232,764 cells labeled by case ID. **b)** Same as A, but cells are labeled by cell type. **c)** scRNA cell type proportions across samples in the cohort. The bigger the circle, the higher the proportion. **d)**. Spearman correlations of tumor estimates. The 95% confidence interval is shown. Top: histology vs scRNA, Middle: ABSOLUTE vs scRNA, Bottom: ABSOLUTE vs histology. **e)** Proteomics and phosphoproteomics PCAs labeled by case ID. **f)** Proteomics and phosphoproteomics PCAs labeled by TMT plex. **g)** Top: Genomic landscape of the cohort showing the top significantly mutated genes. The color scale denotes variant allele fraction (VAF) for each gene. The top bar plot indicates mutation burden for each sample. Bottom: Bulk omics overview of the cohort. The first row indicates germline mutation status followed by scRNA tumor fraction, immune subtype, relative scores of immune and stroma, and different PDAC subtypes (Moffitt, Collisson, Bailey) by piece.

We collected 73 PDAC samples from 21 patients undergoing standard treatment, including four normal adjacent tissue samples. Treatment groups included seven treatment-naïve cases, eight neoadjuvant FOLFIRINOX (a treatment regimen comprising folic acid, 5-fluorouracil, irinotecan and oxaliplatin) cases, four neoadjuvant gemcitabine + nab-paclitaxel cases, one mixed (FOLFIRINOX and gemcitabine + nab-paclitaxel) and one chemoradiation (Chemo-RT) case (Supplementary Table 1). Each tumor was spatially sampled 2–4 times, with sample segments subsequently used to generate histologic, imaging and omics data; hematoxylin and eosin (H&E) slides; scRNA-seq; bulk mass spectrometry-based proteomics and phosphoproteomics; bulk whole-exome sequencing (WES); and bulk RNA-seq (Fig. 1a, Supplementary Table 2 and Methods). We generated scRNA-seq data for all 73 samples, WES for 64 samples and bulk RNA-seq for 65 samples. A subset (*n* = 30) underwent tandem mass tag (TMT) 11﻿ proteomic and phosphoproteomic characterization (Fig. 1b). Following quality control, we clustered 232,764 cells across all samples based on expression profiles and assigned cell types based on marker gene expression (Fig. 1c, Extended Data Fig. 1a–c, Methods and Supplementary Note). Using the fraction of tumor cells as a proxy for tumor purity, estimates ranged from 0.10% to 82.69% across samples, with an average of 16.28%. H&E pathology review revealed that within-patient tumor content differences across samples averaged 24%, with a range of 5% to 64% (Extended Data Fig. 1d, Methods and Supplementary Note), consistent with tumor percentages from scRNA-seq (Pearson *R* = 0.40, *P* = 0.001). Principal component analysis (PCA) of bulk proteomic and phosphoproteomic data confirms that, while most within-tumor regions cluster closely, several specimens from the same tumor have substantial intra-tumor heterogeneity (Extended Data Fig. 1e,f).

We further generated snRNA-seq with matching spatial transcriptomics, RNA-seq and WES data for an additional 10 cases, bringing the total cohort to 31 patients, 83 sc/snRNA-seq samples and 15 spatial transcriptomics slides (Fig. 1d). The treatment groups included three treatment-naïve cases, four neoadjuvant FOLFIRINOX cases, one mixed (FOLFIRINOX and gemcitabine + nab-paclitaxel) and one Chemo-RT case. Following quality control, we assigned cell types to 83,860 nuclei based on marker gene expression and used the paired snRNA-seq to label spots in the spatial transcriptomics slides (Fig. 1d,e and Methods).

### PDAC tumor subclusters with distinct cellular functions

**Fig. 2:**
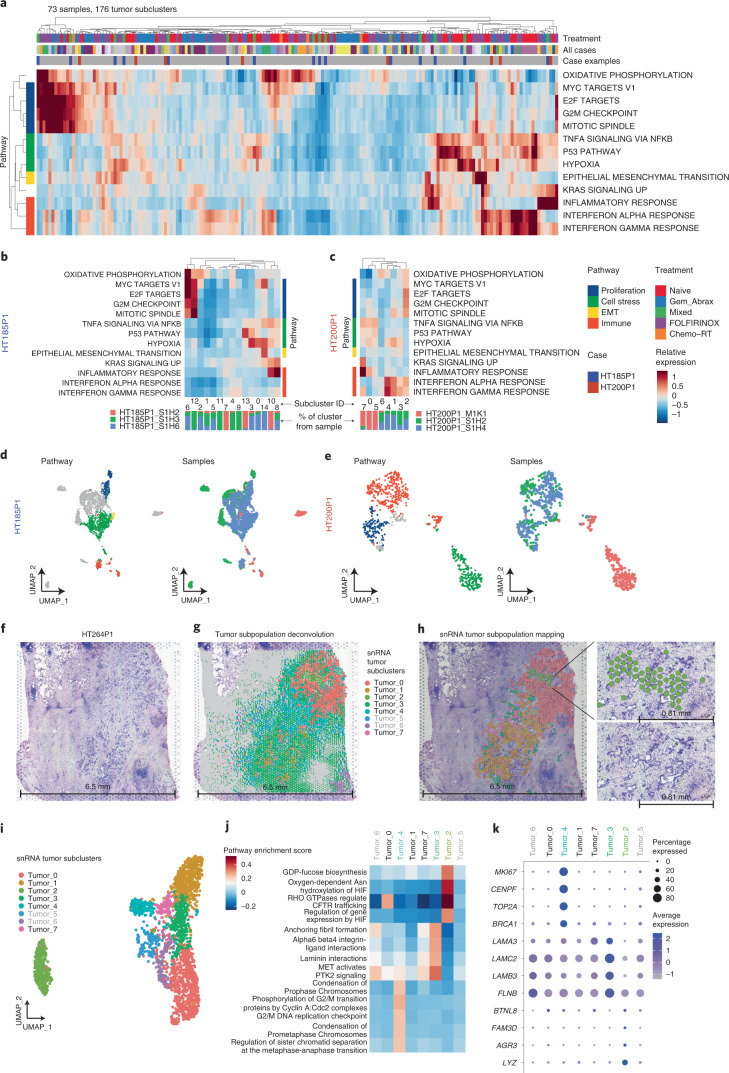
Tumor subclusters with distinct cellular functions. **a**, Differential pathway enrichment case-level tumor subpopulations. Each column is a different tumor subcluster; the top bar indicates treatment and the bottom indicates case ID. Two samples are highlighted: HT185P1 (red) and HT200P1 (blue). The heatmap denotes relative expression of each pathway for each tumor subcluster, grouped by pathway similarity indicated on the left (Methods). **b**, Tumor cluster pathway enrichment for HT185P1. For panels **b** and **c**, each column indicates a tumor subcluster (ID is listed at the bottom of each column), below which is a bar plot indicating the percentage of the cluster that comes from each spatial sample. The heatmap is colored by the relative expression of each pathway. **c**, Tumor cluster pathway enrichment for HT200P1. **d**, UMAP of tumor subclusters for HT185P1. The left UMAP highlights tumor subclusters with high relative expression of associated pathways outlined in panel **b**. The right UMAP indicates tumor subclusters colored by spatial samples. **e**, UMAP of tumor subclusters for HT200P1. UMAPs are equivalent to those shown in panel **d** but for pathways in panel **c**. **f**, H&E of the section used for spatial transcriptomics for HT264P1. **g**, snRNA-seq tumor subcluster mapping using the RCTD deconvolution approach for each spatial transcriptomic spot (Methods). Gray regions in each pie chart denote nontumor cell types and grayed out tumor subpopulations (Tumor_5 and Tumor_6) represent subpopulations with minimal mapping to the H&E section. **h**, Left, simplified version of **g**, where each spot with high confidence was assigned a single tumor subpopulation identity. Right, magnification of the Tumor_2 subpopulation, which has a different morphology from the surrounding tumor. **i**, UMAP of tumor nuclei subclusters from the paired HT264P1 snRNA-seq data. **j**, Pathway enrichment analysis of tumor subclusters focusing on the Tumor_2, Tumor_3 and Tumor_4 subpopulations. **k**, Top DEGs of tumor subclusters focusing on the Tumor_2, Tumor_3 and Tumor_4 subpopulations. The size of each bubble indicates the percentage of cells expressing the gene of interest and color indicates average expression. EMT, Epithelial-mesenchymal transition.

Pathway enrichment analysis between case-level tumor subpopulations to dissect tumor heterogeneity (Fig. 2a and Methods) identified case-specific subpopulations enriched in pathways including cell proliferation, cell stress response, epithelial-to-mesenchymal transition and immune-related pathways that displayed spatial heterogeneity (Fig. 2a and Extended Data Fig. 1g). Actively proliferating clusters were present in most cases and were characterized by upregulation of genes belonging to the Molecular Signatures Database hallmark gene sets^32^ for E2F targets, G2M checkpoint, MYC targets and mitotic spindle. These clusters also exhibited increased oxidative phosphorylation, in line with previous reports^33^ (Fig. 2a–c). It was common (15 of 21 cases) for tumor subclusters enriched in certain pathways to originate predominantly from only one of the spatially distinct samples from each case, such as S1H3 in HT185P1 and S1H4 in HT200P1 (Fig. 2d,e). Other sets of co-upregulated genes included ‘KRAS signaling up’ and ‘inflammatory response’, which lead to pancreatitis, pancreatic intraepithelial neoplasia (PanIN) and eventually PDAC^34^. Increased expression of these sets occurred in samples with lower numbers of proliferating tumor cells, as demonstrated by clusters 7 and 0 from sample HT200P1_M1K1 and cluster 10 from HT185P1_S1H2 (Fig. 2b,c). KRAS-associated inflammatory response was expressed in clusters with increased expression of gene sets associated with cell stress (defined by the TP53 pathway, hypoxia and TNFA signaling via NFKB). This could indicate that parts of the tumor with the most actively proliferating cells were least impacted by KRAS-driven inflammation, or that tumor cells modulate their KRAS-driven associated inflammation during proliferation (Fig. 2d,e and Methods).

Using the spatial transcriptomics cohort, we characterized spatial heterogeneity by integrating histology features. Most slides had dense stroma intermingled with tumor populations (Fig. 1e). For HT264P1, six of eight tumor subclusters were mapped from snRNA-seq data to the spatial transcriptomics spots using cell-type label transfer and Robust Cell Type Decomposition (RCTD) to deconvolve the cell-type composition of each spot (Methods and Fig. 2f,g)^35^. Subpopulation ‘Tumor_2’ clustered separately from other tumor cells and had a distinct ‘lower grade’ morphology (as annotated by pathology) (Fig. 2g–i). Gene and pathway enrichment analysis revealed that the ‘Tumor_2’ population upregulates fucosylation, hydroxylation and HIF pathways, and genes associated with the basal-like tumor subtype (*BTNL8*, *AGR3* and *LYZ)* (Fig. 2j,k). These findings suggest that this population is a spatially distinct cluster with different H&E morphology and likely more aggressive, highlighting the heterogeneous composition of tumor cells in PDAC in terms of pathology and transcriptomics. We identified ‘Tumor_4’ as a proliferative population that upregulates cell cycle pathways (Fig. 2j,k). Lastly, we characterized ‘Tumor_3’, which is scattered around the periphery of the large tumor regions in H&E (Fig. 2g,h). This population has high expression of laminin genes and is enriched in integrin and fibril formation pathways, which interact with the extracellular matrix and may be involved in tumor expansion (Fig. 2j,k)^36^. In summary, we observe spatially and transcriptionally distinct tumor subpopulations from the same patient sample, within the same H&E section.

### KRAS signaling and spatial drivers in pancreatic cancer

**Extended Data Fig. 2:**
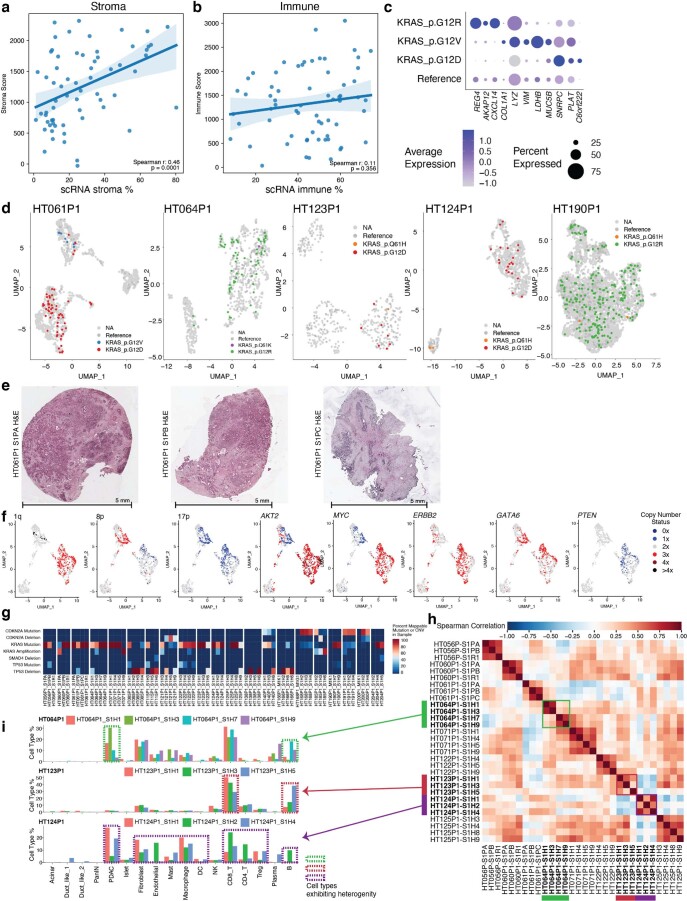
Genomic Features in Heterogeneous KRAS Subpopulations. **a)** Spearman correlation of scRNA estimates and ESTIMATE stroma score. **b)** Spearman correlation of scRNA estimates and ESTIMATE immune score. For panels A and B, the 95% confidence interval is shown. **c)** Top significant DEGs between specific *KRAS* hotspot mutations. Only cells with a mappable *KRAS* mutation were included in this analysis. **d)** *KRAS* mutations in tumor cells of 5 cases with multiple *KRAS* variants mapped. **e)** H&E images of the spatial samples in HT061P1. **f)** Arm and gene-level CNV events in HT061P1 mapped to different tumor clusters. **g)** Percent of mappable mutations or deep copy number amplifications and/or deletions in each sample for KRAS, TP53, CDKN2A, and SMAD4. Samples are grouped together by case and cases are separated by white lines. **h)** Protein-level pairwise spearman correlation between all 30 samples that underwent bulk proteomics. Boxed cases represent cases with high heterogeneity: HT064P1 in green, HT123P1 in red, and HT124P1 in purple. **i)** Cell type proportion distribution of the three heterogeneous cases from panel H. Cell types in dotted boxes represent substantial differences in cellular composition that likely underlie the observed heterogeneity.

We observed substantial variation of driver mutation variant allele fractions (VAFs) between samples (Extended Data Fig. 1g and Supplementary Data 1). We detected a *KRAS* hotspot mutation in at least one sample in all cases, except for HT138P1 (HT204P1 lacks WES data). For low VAF (<0.01) mutations, 10 of 20 cases had samples whose mutation profiles differed from one another. We also identified pathogenic germline variants^37^ (Methods). HT138P1 carried a pathogenic germline *BRCA2* variant. Three cases carried variants in the homology-directed DNA repair pathway (*FANCC* D23*, *BRCA2* I1470* and K607*, and *ATM* Y1124*), and, expectedly, all spatial samples carried the same variant in each case. Using RNA-seq, we classified samples into established subtypes^12,38,39^ and determined immune subtypes and stromal and immune compartment scores using xCell^40^ and ESTIMATE^41^, respectively (Extended Data Figs. 1g and 2a,b, Supplementary Note and Methods).

**Fig. 3:**
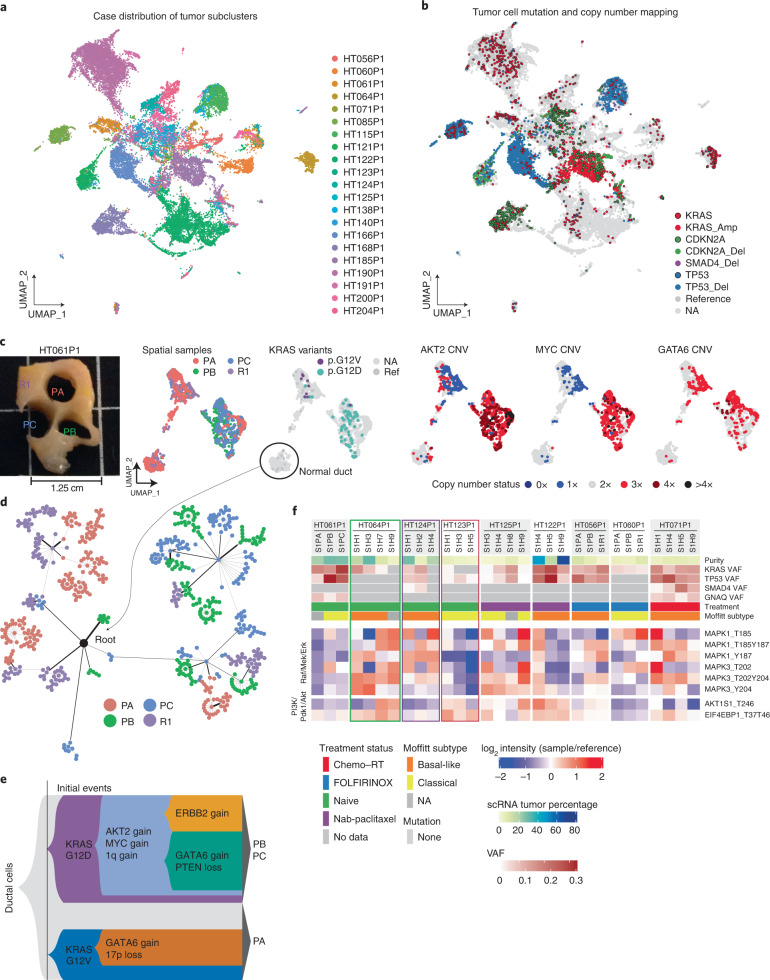
Genomic landscape and oncogenic driver heterogeneity. **a**, Tumor cell clusters labeled by case ID. **b**, Tumor cells labeled with genomic alterations including mutations and copy number alterations. Mutations are denoted by colored circles with a black outline, while deep copy number events (gain/loss of more than 1 copy) are denoted by colored circles without an outline. **c**, Ductal cells from case HT061P1. From left to right, tissue sample spatial locations; spatial sample IDs (R1 denotes the remainder tissue); KRAS variant mapping; and *AKT2* CNV, *MYC* CNV and *GATA6* CNV mapping. Spatial samples are labeled with a ‘P’ to denote punches and ‘R’ to denote remainder tissue. Copy number calls were obtained using inferCNV and CNV status is indicated by color. **d**, CNV-based lineage tree of a subset of ductal cells from HT061P1. **e**, Proposed model of tumor progression for HT061P1. **f**, Bulk phosphosite levels in the PI3K/Pdk1/Akt and Raf/Mek/Erk pathways. Cells filled in gray denote missing data. Samples with proteomics/phosphoproteomics all did not have mutations in *CDKN2A*. NA, No coverage for mutant or reference allele; Ref, Reference allele.

Tumor cells grouped into patient-specific clusters, consistent with the genomic landscapes within each patient (Fig. 3a and Methods). We mapped mutation and copy number alterations to single cells (Fig. 3b and Methods). We tested the impact of *KRAS* hotspot variants by comparing gene expression profiles of each subset of tumor cells with a given *KRAS* mutation (Methods). Interestingly, tumor cells harboring *KRAS* G12V upregulate several genes associated with more aggressive or metastatic tumors, including *COL1A1*, *VIM* and *MUC5B* (Extended Data Fig. 2c)^42–44^. We identified five cases with multiple *KRAS* hotspot drivers, which we interpret as synchronous primary tumor clones^11,45,46^ (Extended Data Fig. 2d). For case HT061P1, we obtained four subpopulations of clustered tumor cells, three small clusters largely derived from punch A and one large cluster common to all three punches (the remainder, as expected, represented all clusters) (Fig. 3c and Extended Data Fig. 2d,e). *KRAS* G12V cells faithfully map into one cluster from punch A predominant populations, with G12D cells mapping onto the large mixed cluster. Thus, two distinct clones carrying different *KRAS* driver mutations in the same patient are spatially separated, with differing gene expression profiles (Fig. 3c).

Using inferCNV^47^, we identified copy number variation (CNV) signatures at both focal and arm levels that are unique to the respective *KRAS* subclones in case HT061P1 (Fig. 3c, Extended Data Fig. 2f and Methods). Copy number alterations were further evaluated using WES (Supplementary Data 2 and Methods). The G12D population shows amplifications of *AKT2* and *MYC*, while both G12D and G12V clusters harbor amplifications in *GATA6*, among others (Fig. 3c and Extended Data Fig. 2f). We reconstructed a lineage tree using MEDALT^48^ (Fig. 3d and Methods), separating tumor cells into two major groups, each branching from a normal duct origin, consistent with both gene expression-based clustering and spatial origin of the cells (Fig. 3c,d). Together, we propose a model that integrates the gene expression and CNV data (Fig. 3e and Supplementary Note).

To assess proteomic heterogeneity, we conducted a global pairwise correlation analysis for the *n* = 30 samples from 9 tumor cases (Methods and Supplementary Note). We determined the impact of mutations on downstream targets by analyzing the associated changes at protein and phosphorylation levels in several oncogenic pathways (Methods). We found a large degree of differential regulation, between and within tumors, in several phosphosites within the PI3K/PDk1/Akt and Raf/Mek/Erk pathways (Fig. 3f, Extended Data Fig. 2g–i and Supplementary Note).

### Transitional populations between acinar and tumor cells

The hypothesis that PDAC arises from acinar cells that undergo ADM^49–51^ has been examined in mice, but not humans^52^. A major hurdle has been the small numbers of acinar and ADM cells that have been sampled from patients at single-cell resolution^24,25^.

**Fig. 4:**
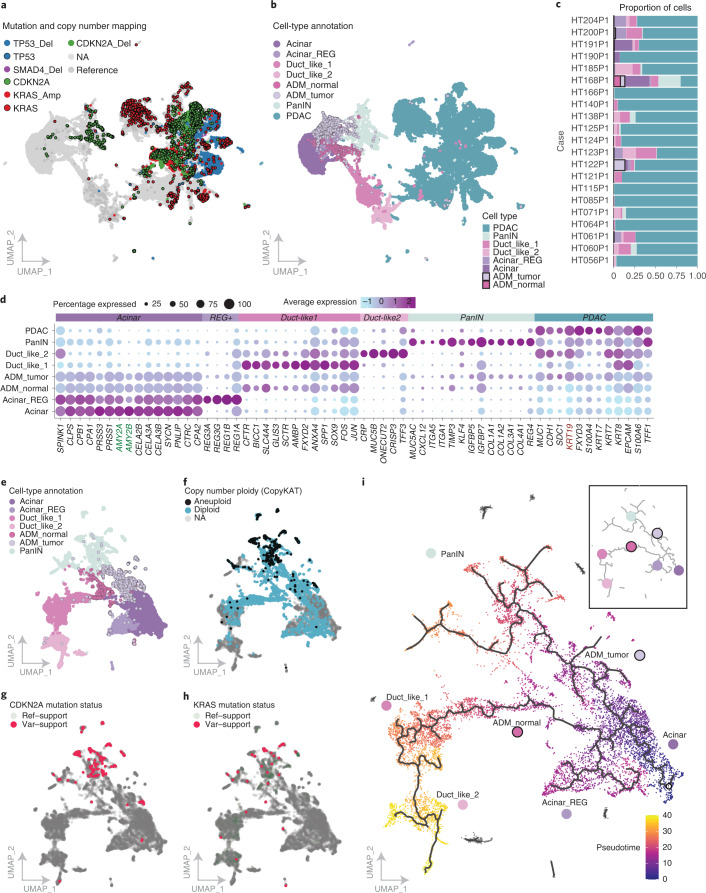
Acinar, ductal and transitional populations. **a**, UMAP clustering of acinar, ductal, transitional and PDAC tumor cells across the single-cell cohort. Mutations and deep copy number events were mapped to individual cells (Methods). Copy number events are indicated by colored dots with no outline, while an outlined black circle denotes a mutation event. **b**, UMAP clustering of acinar, ductal, transitional and PDAC tumor cells colored by cell-type annotation from single-cell RNA-seq samples. **c**, Proportion of cells identified as acinar, ductal, transitional and PDAC tumor cells by sample. **d**, Highly expressed genes identified in each cell type. The size of the bubble indicates the percentage of cells expressing the gene of interest and color indicates average expression. The bubble plot is ordered by expression across each cell-type group. *AMY2A*/*2B* and *KRT19* are indicated in bold because they are genes used for staining acinar cells and ductal cells, respectively, in the following immunofluorescence assays. ADM cells show expression of both of these markers in the scRNA data. **e**, Cell-type annotation and genomic alterations mapped across acinar, normal ductal, PanIN and ADM populations. UMAP of cell-type annotation indicates two distinct ADM populations annotated as ADM_Normal and ADM_Tumor. **f**, Copy number was annotated using CopyKAT, which predicts aneuploid cells independent of identifying tumor populations. Cells are colored by ploidy status. **g**,**h**, CDKN2A (**g**) and KRAS (**h**) mutation mappings are indicated in the lower two UMAPs and are colored by reference, variant and variant/reference supporting cells. **i**, Monocle pseudotime analysis indicates a cell-state transition from acinar cells to ADM_Tumor and ADM_Normal states independently. Each cell is colored by pseudotime which is a measurement of the change each cell is making through a process (for example, differentiation) and is annotated with a trajectory of change in the solid line overlaying the UMAP. Inset of the trajectory shows a summary of the cell-state transitions, with dots indicating cell type.

**Extended Data Fig. 3:**
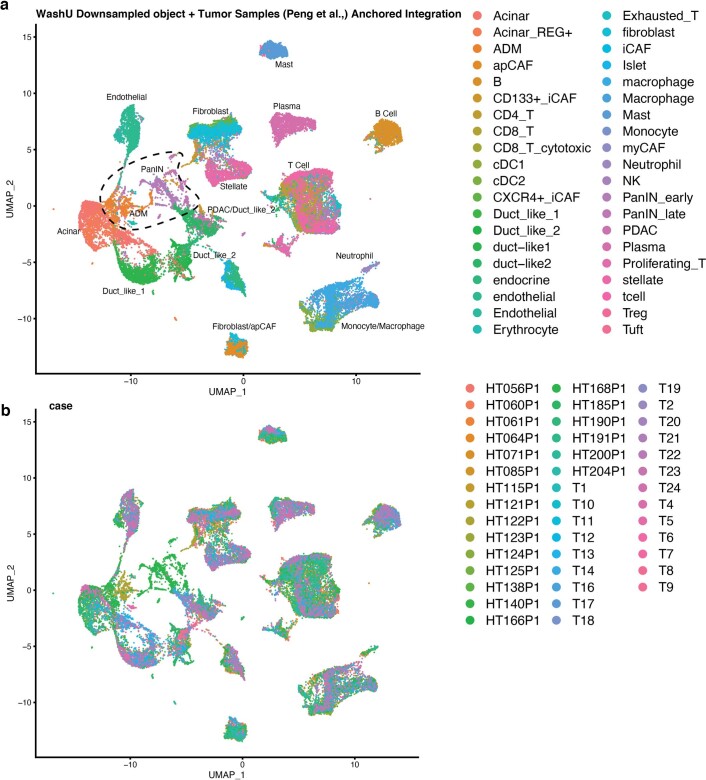
Evaluating Transitional Populations in Published Studies. **a)** Integration of downsampled cells from tumor samples from Peng et al.^24^, and WashU samples. UMAP shows integrated single cells colored by cell type. Circled region indicates cells that are specific to the WashU Cohort predominantly made of up PanIN and ADM identified cells. **b)** Integration of downsampled cells from tumor samples from Peng et al., and WashU samples. UMAP shows integrated single cells colored by case. Cases indicated with ‘HT#P1’ are WashU samples while samples with ‘T#’ are from Peng et. al.

We identified populations of acinar cells expressing acinar markers (*PRSS1*, *CELA3A*) from multiple samples (Fig. 4a–d). The Acinar-REG^+^ cluster exhibits high expression of regenerating proteins^25,30,53,54^ thought to promote ADM and PanIN in PDAC^55,56^. Two mixed populations of ductal cells^30,54^ lacked genomic alterations and maintained high expression of ductal markers (*CFTR*, *SLC4A4*, *ANXA4*, *SOX9*). Duct-like1 expresses *SPP1* and *CRP*, which have been observed in stressed cells and have progenitor-like features from the pancreatic ductal niche^25^. Duct-like2 expresses normal ductal genes to a lesser extent and shows increased expression of mucus secretion (*MUC5B*) and trefoil factor genes. Highly expressed markers in Duct-like2 suggest these cells are a major source of malignant PDAC cells^24^, and transcriptionally resemble cells identified in healthy pancreases^30^. Finally, this cluster exhibits expression of *ONECUT2*, a transcription factor exclusively expressed in metaplastic cells derived from acinar origin in a mouse model^54^. Peng et al.^24^ hypothesized that subclusters of Duct-like2 could be PanIN-like, but we find that our PanIN and ADM populations are distinct from Duct-like2 and are only identified in our cohort (Extended Data Fig. 3a,b and Supplementary Note). PDAC exhibits high expression of *FXYD3*, *S100P* and *KRT17* in addition to copy number and driver mutations. PanIN-like cells were derived from 19 patients with a large proportion from sample HT168P1 (>1,700 cells) and, consistent with previous studies, we observe PDAC-initiating mutations in *KRAS* and *CDKN2A* (from HT168P1) within PanIN populations^52^. PanIN exhibits increased expression of extracellular matrix-related genes (*DCN*, *SPARC*, *SPON1*), a diversity of collagens^57^, genes involved in acinar-to-ductal reprogramming (*KLF4*, *MMP7*)^58,59^ and other markers of early-stage malignancy (*CXCL12*, *TIMP3*, *ITGA1*, *MUC5AC*)^60–63^.

We detected ADM cells in eight samples, including from cases HT122P1 and HT168P1 that represented a large proportion of ADM cells (>700 cells) (Supplementary Data 3). We found PDAC cells harboring several distinct alterations (Fig. 4a,b). Cells expressing acinar and ductal markers clustered separately from acinar cells and tended to be between acinar cells and normal ductal lineages on the Uniform Manifold Approximation and Projection (UMAP) (Duct-like1, Duct-like2), termed ADM_Normal, or between acinar and PanIN, denoted ADM_Tumor (Fig. 4b,c). While tumor and acinar cells express ductal and acinar markers in a mutually exclusive pattern, ADM cells express a combination, suggestive of an intermediate, reversible state (Fig. 4d). While both ADM_Tumor and ADM_Normal have decreased expression of acinar markers, they also have increased expression of PDAC markers and Duct-like1 markers, respectively. Following copy number analysis (CopyKAT, Methods), we found that a majority of predicted aneuploid cells are annotated as PanIN (*n* = 524), while only a handful (*n* = 30) in ADM_Tumor are labeled aneuploid (Fig. 4e,f). By mapping both *KRAS* and *CDKN2A* mutations, we identified several cells in the ADM_tumor population with either a *KRAS* mutation (*n* = 1) or *CDKN2A* mutation (*n* = 7), although this was not as widespread as the predicted PanIN populations (KRAS: 23 cells; CDKN2A: 163 cells) (Fig. 4g,h and Supplementary Data 3).

We examined whether acinar cells transition to different expression states (tumor or normal) by way of the two distinct ADM cell populations by performing Monocle analysis (Methods). We found two different transition states starting either with acinar cells transitioning towards the normal ductal cell route with ADM_Normal cells in between or with cells transitioning from acinar cells towards PanIN cells with ADM_Tumor cells in between (Fig. 4i). This suggests ADM_Normal is a transition state more similar to normal ductal cells and largely lacking genomic alterations, while ADM_Tumor is more related to PanIN and has a few alterations (for example, *CDKN2A*, aneuploidy). Recent studies in mice suggest acinar-derived tumors are preceded by PanINs, while ductal-derived tumors are PanIN independent^64^.

### Validation of ADM using snRNA-seq, immunohistochemistry and mouse models

**Fig. 5:**
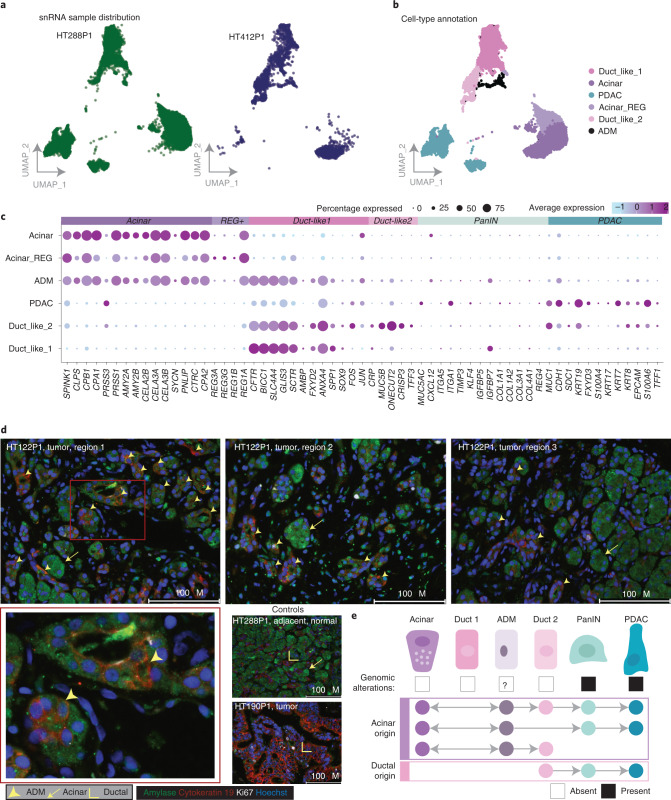
Validation of ADM using snRNA and immunofluorescence. **a**, UMAP plots of acinar and ductal cells from two cases, HT288P1 and HT412P1. Cells are colored by sample. **b**, UMAP plots of acinar and ductal cells colored by cell types. **c**, Gene expression signatures derived from scRNA data of acinar and ductal genes across cell types. Each dot indicates expression of a given gene in an annotated cell cluster. The size indicates the percentage of cells expressing that gene and the color is average expression. ADM cells show expression of both acinar and ductal markers in the snRNA data. **d**, Immunofluorescence staining of tumor and NAT sections. Amylase stains acinar cells (green), cytokeratin-19 stains ductal cells (red), Ki67 stains proliferating cells (white) and Hoechst stains nuclei (blue). For select sections, individual cells expressing both acinar and ductal markers, indicating ADM, are highlighted by the yellow arrowheads. Acinar cells are denoted with a yellow arrow and ductal cells with an outlined yellow angle. **e**, Proposed models of PDAC development. Development of PDAC along the spectrum from normal to PDAC in humans was initially suggested to be derived predominantly from ductal origin, but, with evidence of ADM cells in humans, an additional model of transition from acinar origin to PDAC is proposed.

We orthogonally surveyed two samples by snRNA-seq to see if cells expressing acinar and ductal features could be identified from frozen tissue (Fig. 5a–c). ADM cells in HT288P1 and HT412P1 snRNA-seq samples have higher expression of Duct-like1 features than tumor cells, suggesting similarity to the ADM_Normal population in scRNA-seq.

**Extended Data Fig. 4:**
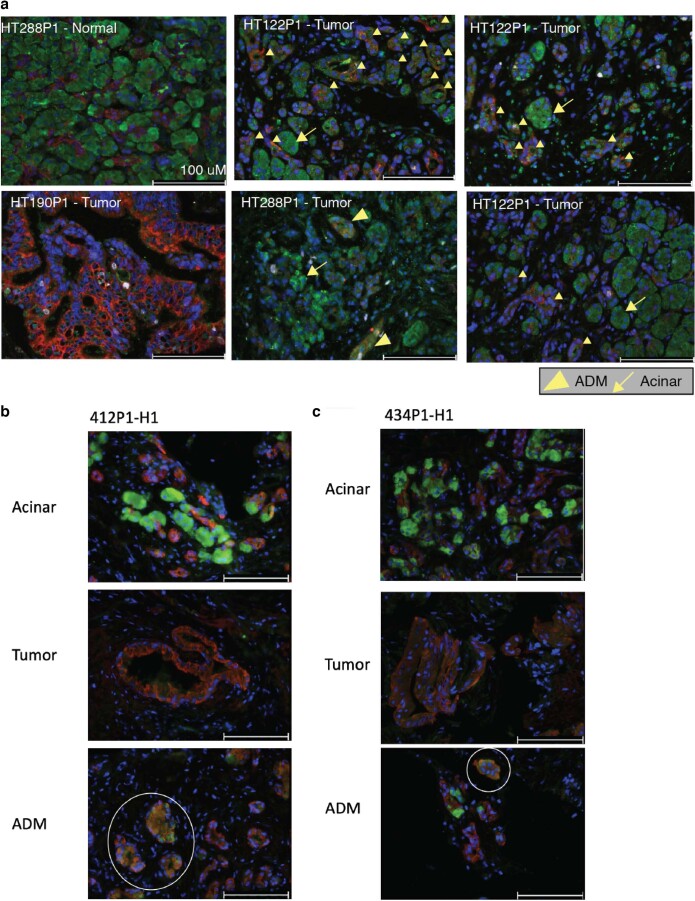
Combined Channel Immunofluorescence Images of ADM Samples. **a)** Combined channel immunofluorescence staining across four samples HT288P1 (Adjacent Normal), HT190P1 (Tumor), HT122P1 (Tumor) and HT288P1 (Tumor). Amylase stains acinar cells (green), cytokeratin 19 stains ductal cells (red), Ki67 stains proliferating cells (white), and Hoechst stains nuclei (blue). For select sections, individual cells expressing both acinar and ductal markers indicating acinar to ductal metaplasia (ADM) are highlighted by the yellow triangle. Acinar cells are denoted with a yellow arrow. **b)** Combined channel immunofluorescence staining across two samples HT412P1 (Tumor) and **c)** HT434P1 (Tumor). Amylase stains acinar cells (green), cytokeratin 19 stains ductal cells (red), Ki67 stains proliferating cells (white), and Hoechst stains nuclei (blue). Cells exhibiting co-expression of Amylase and cytokeratin 19 are circled in white. Regions of the section with high acinar content, tumor content and ADM content are shown from top to bottom for each sample.

As validation, we performed immunofluorescence staining on tumor and normal formalin-fixed paraffin-embedded (FFPE) sections with amylase (acinar), cytokeratin-19 (ductal), Hoechst (nuclei) and Ki67 (proliferation) to evaluate co-staining patterns within individual cells (Fig. 5d, Extended Data Fig. 4a and Methods). In HT122P1 and HT288P1, we observe co-staining of acinar and ductal markers within multiple individual cells across several tumor regions. As controls, we provide a normal section with intermixed acinar and ductal cells, but lacking a co-staining expression pattern (HT288P1), and a tumor section that is predominantly stained by cytokeratin-19 (HT190P1). The paucity of ADM using immunofluorescence was recently validated in a tamoxifen-induced PDAC mouse model where acinar transformed ductal cells similarly co-stained for amylase and Cytokeratin-19 (ref. ^65^). Although rare, this co-staining pattern is confirmed at single-cell resolution in the same samples for which we performed immunofluorescence, thanks to our spatial sampling strategy. We identified two additional samples (HT412P1, HT434P1) with high acinar content with the same co-expression patterns (Extended Data Fig. 4b,c).

**Extended Data Fig. 5:**
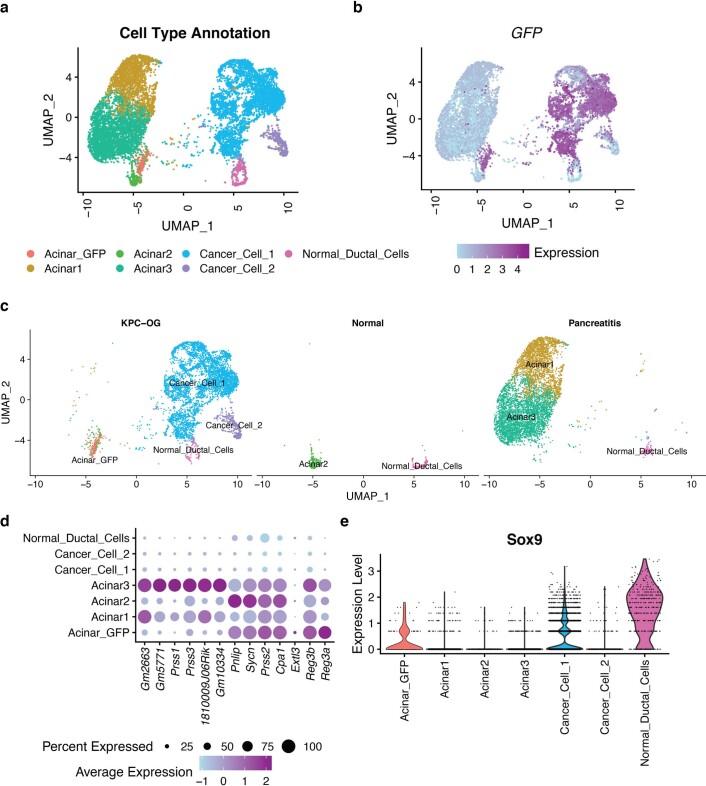
Single Cell Analysis of Mouse Model Validating Transitional Acinar Populations. **a)** UMAP of acinar and ductal single cells from mouse model. Cells are colored by cell type. **b)** UMAP of GFP expression. Cells are colored by expression value. **c)** UMAP of acinar and ductal cells separated by mouse model from which cells are derived from. **d)** Selected gene expression markers of acinar and ductal genes across cell types. Each dot indicates expression of a given gene in an annotated cell cluster. The size indicates the percent of cells expressing that gene and the color is average expression. **e)** Violin plot showing the distribution of expression levels of Sox9 across each annotated cell type.

Finally, we performed scRNA-seq on eight mice from mouse models including induction of pancreatitis, *KRAS-*driven early- and late-stage transformation (KPC-OG GEMM mice) and normal pancreas tissues (Methods)^66^. We identified two pancreatitis-acinar populations, one tumor-acinar population and one normal-acinar (Extended Data Fig. 5a–c). Markers within the pancreatitis-acinar populations overlap differentially expressed genes (DEGs) identified in another study^67^ (Extended Data Fig. 5d). The tumor-acinar population from the KPC-OG model was the only acinar cluster with GFP expression (Extended Data Fig. 5b). Within this tumor model, GFP is associated with early transformation and metaplasia. While the tumor-acinar population expresses *Reg3a*, which is overexpressed in ADM regions^68^, it also maintains high expression of *Sox9*, a ductal lineage marker in normal ductal and cancer cells, suggesting early-stage metaplasia.

These results support the identification of this rare population with acinar and ductal-like features seemingly lacking widespread genomic alterations. Our findings enable us to expand the proposed models of acinar origin to human PDAC development (Fig. 5e).

### Transitional populations in histological features by spatial transcriptomics

**Fig. 6:**
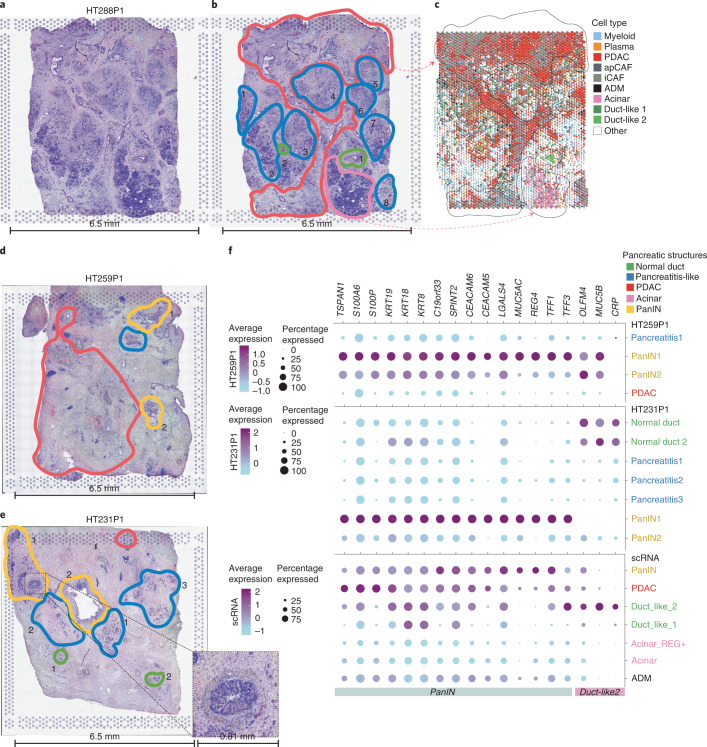
Tumor and transitional cell heterogeneity in spatial transcriptomics data. **a**, H&E of the section used for spatial transcriptomics for HT288P1. **b**, Pathologist-annotated regions of HT288P1. Regions highlighted include tumor (red), pancreatitis-like (blue), normal duct (green) and acinar (pink). Numbers are listed next to each annotated area if more than one are reported. **c**, snRNA-seq mapping using the RCTD deconvolution approach for each spatial transcriptomics spot (Methods). Each spot is colored by the expected representation of the cell types discovered from snRNA-seq. Regions of tumor, normal duct and acinar are highlighted with black dashes, showing similarity between pathology-assisted annotations and deconvolution. **d**, Pathologist-annotated regions of HT259P1. Numbers are listed next to each annotated area if more than one is reported. **e**, Pathologist-annotated regions of HT231P1. Numbers are listed next to each annotated area if more than one is reported. **f**, DEGs identified by spatial transcriptomics pathology-assisted regions with a focus on normal duct and PanIN. The size of the bubble indicates the percentage of cells expressing that target gene and color indicates average expression. Top, HT259P1 spatial transcriptome DEG analysis. Middle, HT231P1 spatial transcriptome DEG analysis. Bottom, spatial transcriptome-derived DEGs mapped to scRNA-seq acinar, ductal and transition populations.

**Extended Data Fig. 6:**
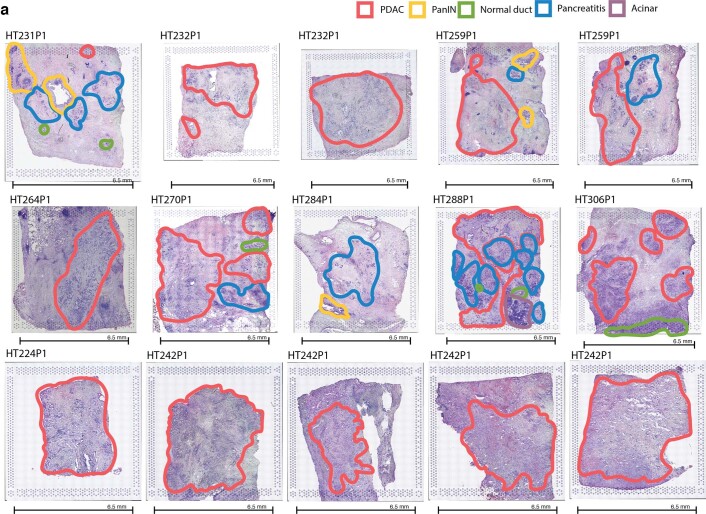
Evaluating Transitional Populations with Spatial Transcriptomics and Published Studies. **a**) H&E images associated with each piece of tumor that underwent spatial transcriptomics processing. Regions on slides are highlighted based on pathology assisted review. Regions are indicated as tumor (Red), PanIN (Yellow), Normal Duct (Green), Pancreatitis (Blue) and Acinar (Purple).

Spatial transcriptomics enables the identification of histology features (for example, PanIN) to complement findings in sc/snRNA-seq. Each H&E image with associated spatial transcriptomics data was annotated by a pathologist (Extended Data Fig. 6a). Of ten paired spatial transcriptomics/snRNA-seq samples, HT288P1 presented with ADM (88 cells). We compared pathology annotations with spots defined via integration with snRNA-seq and with RCTD (Fig. 6a–c)^35^. We observe strong concordance between pathology-defined and molecular regions for tumor, normal duct and acinar cells. Myeloid and plasma cells mapped throughout annotated pancreatitis regions, Duct-like2 mapped to annotated normal ductal structures and ADM mapped sparsely throughout the section. It remains difficult to validate ADM in spatial transcriptomics data since it has not yet reached single-cell resolution and ADM cells are rare. Under these circumstances, pancreatitis regions may appear to have both acinar and ductal features in agreement with Tosti et al.^30^.

We annotated spatial transcriptomics spots in select samples with PanIN and a ductal structure within the capture area (Fig. 6a–e). Extracted spots were then subsetted from the full object and DEGs were analyzed. Isolated PanIN regions exhibited distinct DEGs that spanned multiple samples (Fig. 6f and Supplementary Data 4). DEGs identified with spatial transcriptomics data for normal duct, tumor and PanIN were compared against annotated scRNA-seq data, corroborating our initial annotations of PanIN, normal duct and tumor. Interestingly, the two cases having multiple PanIN regions identified by spatial transcriptomics exhibited distinct DEGs that differed by each uniquely annotated region. Our combined analysis strongly supports the presence of PanIN and existence of ADM in human samples.

### CAF subpopulations in PDAC TME

**Fig. 7:**
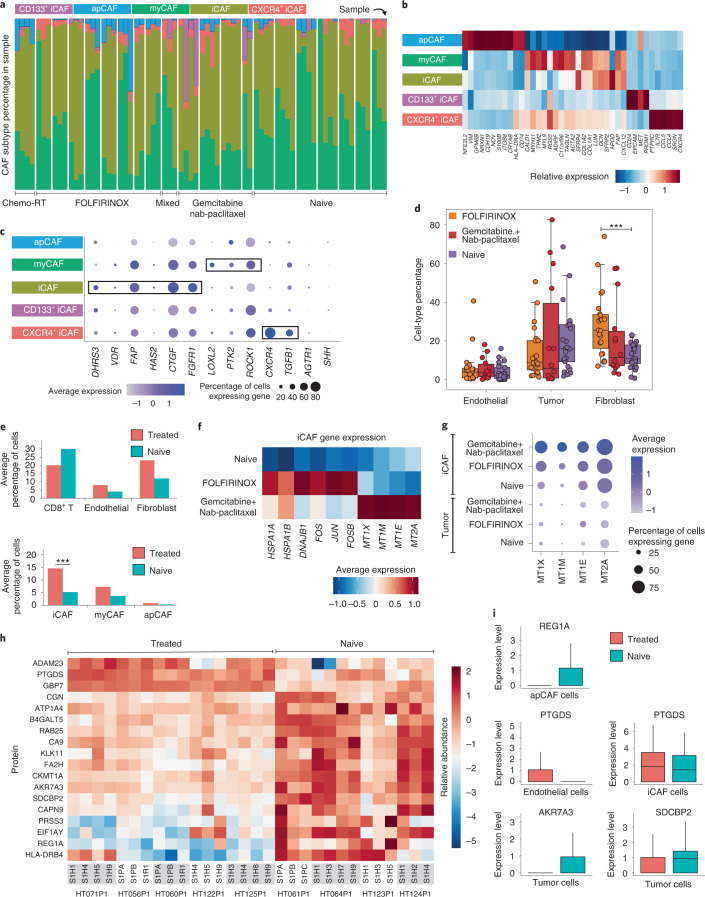
CAF subpopulations across treatment groups. **a**, CAF subtype distribution across the cohort and across treatment groups. CAF subtype colors are indicated at the top and are consistent throughout panels **a**–**c**. **b**, Key markers and DEGs in each CAF subtype. **c**, Expression of genes currently targeted by clinical trials across CAF subtypes. The size of the bubble indicates the percentage of cells expressing the gene of interest and color indicates average expression. **d**, Cell-type percentage differences in tumor, endothelial and fibroblast cells among treatment groups. The mixed and Chemo-RT singleton cases were excluded in these analyses (*n* = 41 treated, *n* = 25 untreated). ***P < 10^−3^ (exact *P* = 0.0026360), using a two-sided Wilcoxon rank sum test. The boxplots show the median with 1.5 × interquartile range whiskers. **e**, Top, average cell-type percentages split into treated versus untreated groups. Bottom, average cell-type distributions of the main CAF subtypes (iCAF, myCAF and apCAF) split into treated versus untreated groups (*n* = 41 treated, *n* = 25 untreated). ***P < 10^−3^ (exact *P* = 0.0065984), using a two-sided Wilcoxon rank sum test. **f**, DEGs between treated and untreated iCAFs. **g**, Expression of metallothionein genes across treatment groups in iCAFs and tumor cells. **h**, Top differentially expressed proteins across treated and untreated samples. **i**, Differential gene expression in specific cell types that match the proteins in panel **h** (*n* = 41 treated samples, *n* = 25 untreated samples). The boxplots show the median with 1.5 × interquartile range whiskers.

**Extended Data Fig. 7:**
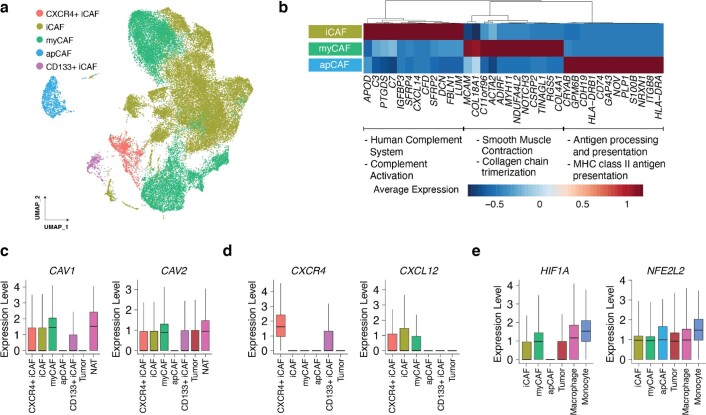
CAF Subtypes. **a)** UMAP of all fibroblast cells labeled by CAF subtype. **b)** Top DEGs and pathways across iCAFs, myCAFs, and apCAFs. **c)** *CAV1* and *CAV2* expression in CAF subtypes, tumor cells, and fibroblasts from NAT samples. **d)** *CXCR4* and *CXCL12* expression in CAF subtypes and tumor cells. **e)** *HIF1A* and *NFE2L2* expression in CAF subtypes, tumor cells, macrophages, and monocytes. FDR < 0.0001 for macrophage and monocyte upregulation of *NFE2L2* and *HIF1A*. Panels D-F include expression from all cells in the study of the given cell type and the boxplots show the median with 1.5x IQR whiskers.

We identified three subtypes of CAFs: myofibroblastic CAFs (myCAFs), inflammatory CAFs (iCAFs) and antigen-presenting CAFs (apCAFs)^14,15,69^ (Fig. 7a and Extended Data Fig. 7a). We also observed subpopulations of iCAFs, denoted as CXCR4^+^ iCAFs and CD133^+^ iCAFs. Several CAF markers, including *ACTA2* and *FAP*, used to identify CAF subtypes are not definitive, being often expressed in both iCAFs and myCAFs^70^. We identified the top DEGs between each CAF subtype; *TAGLN* and *ACTA2* discern myCAFs, *FAP* and *CXCL12* distinguish iCAFs, and apCAFs express *HLA-DRA* and *CD74* (refs. ^14,71^) (Fig. 7b and Extended Data Fig. 7b). CXCR4^+^ iCAFs and CD133^+^ iCAFs are defined by *CXCR4* and *CD133* (*PROM1*), respectively, although they also weakly express myCAF and apCAF marker genes. While most CAFs in tumors are iCAFs or myCAFs, the other CAF subtypes are present at low numbers throughout. These CD133^+^ iCAFs carry no genomic alterations, but express cancer stem cell markers, including *CD133*, *MET*, *EPCAM*, *CD24* and *CD44*. We observed high *CD44* expression in apCAFs and CXCR4^+^ iCAFs. *VIM* and *NFE2L2* were highly expressed in apCAFs, which were more abundant in treated samples (*P* < 10^−5^) (Fig. 7b). These results suggest that small unique CAF subpopulations that express cancer-driving programs exist within standard CAF subtypes. We examined expression of CAF genes currently targeted by clinical trials registered since January 2020 (ref. ^15^) (Fig. 7c). As treated samples have a depletion of myCAFs and enrichment of iCAFs, the effectiveness of additional therapies targeting CAFs may differ across treatment groups. Further, tumor-specific CAF clusters (relative to normal adjacent tissue) were enriched for TME-remodeling pathways (Extended Data Fig. 7c–e and Supplementary Note).

By assessing cell-type enrichment across treatment groups, we detected modest changes in endothelial and tumor cells and the largest difference within fibroblasts, where both treated groups had higher numbers than the treatment-naïve group (Fig. 7d). This is largely driven by a threefold higher level of iCAFs in FOLFIRINOX and Gemcitabine + nab-paclitaxel samples (*P* < 10^−3^), with comparable myCAF abundance between treatment groups (Fig. 7e). As iCAFs are considered to be pro-tumorigenic^72^, this large increase of iCAFs after treatment may be associated with treatment resistance. We observed upregulation in heat shock genes, AP-1 pathway genes and metallothionein genes in treated iCAFs (Fig. 7f). As we only observe substantial expression of metallothionein genes in iCAFs, their prognostic value for predicting chemotherapy resistance originates from the stroma, rather than tumor cells^73^ (Fig. 7g). Heat shock and AP-1 genes were more highly expressed in FOLFIRINOX samples, while metallothioneins were more highly expressed in Gemcitabine + nab-paclitaxel samples, suggesting iCAF heterogeneity based on treatment regimen (Fig. 7f). These observations suggest that treated tumors have much higher levels of iCAFs, which are potential targets for chemo-resistant tumors.

We identified 18 proteins having at least a twofold or greater change between treated and untreated samples (Methods and Supplementary Note). GBP6, PTGDS and ADAM23 were elevated in treated samples, while REG1A, EIF1AY, PRSS3 and HLA-DRB4 were elevated in naïve samples (Fig. 7h). While these proteins display overall differences between treated and naïve samples, we observe modest heterogeneity between spatial samples and between a subset of tumor cases (Methods). To assess whether these proteins are signals originating from tumor cells or the TME, we compared their expression profiles in scRNA data within each cell type among treatment groups. *REG1A* is upregulated in naïve apCAFs, while *PTGDS* is upregulated in treated endothelial and iCAF cells; neither were observed in tumor cells. (Fig. 7i). Only *AKR7A3* and *SDCBP2* were consistently upregulated strictly in tumor cells. These results suggest that several of these differentially abundant proteins may originate from the TME.

### Immunosuppressive PDAC TME and treatment

**Fig. 8:**
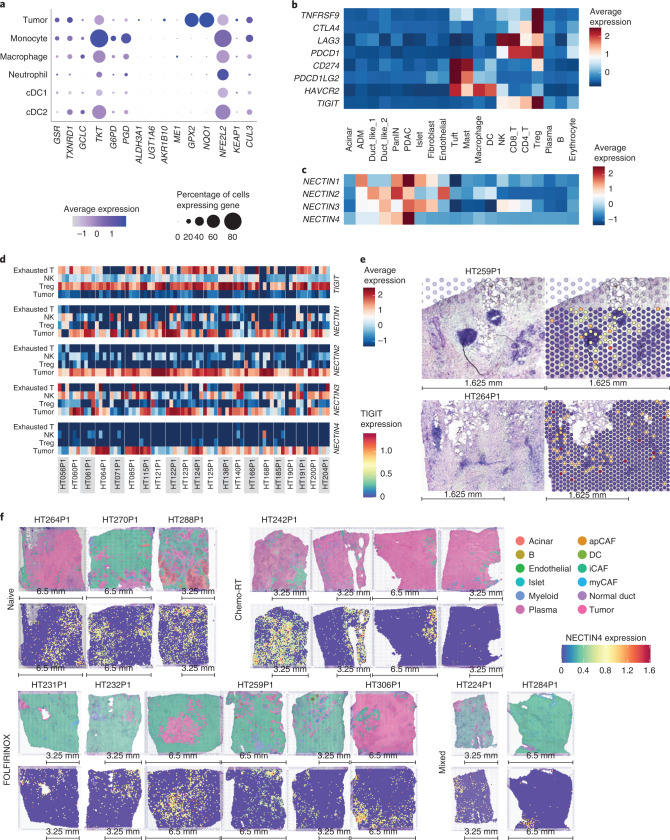
Myeloid and lymphocyte populations in the TME. **a**, Expression of Nrf2 pathway genes in myeloid and tumor cells. The size of the bubble indicates the percentage of cells expressing the gene of interest and color indicates average expression. **b**, Expression of immune checkpoint receptor and ligand genes across cell types. **c**, Expression of the four nectin receptors across all cell types. **d**, Average expression of *TIGIT*, *NECTIN1*, *NECTIN2*, *NECTIN3* and *NECTIN4* in exhausted T cells, NK cells, Tregs and tumor cells. Each column denotes a spatial sample and columns are grouped by case ID. **e**, TIGIT expression in lymphocyte-infiltrated regions from two spatial transcriptomics cases. **f**, *NECTIN4* expression colocalization with tumor spots across the spatial transcriptomics cohort. For each slide, the bottom image shows TIGIT expression and the top shows cell types. Tumor cells are colored bright pink.

**Extended Data Fig. 8:**
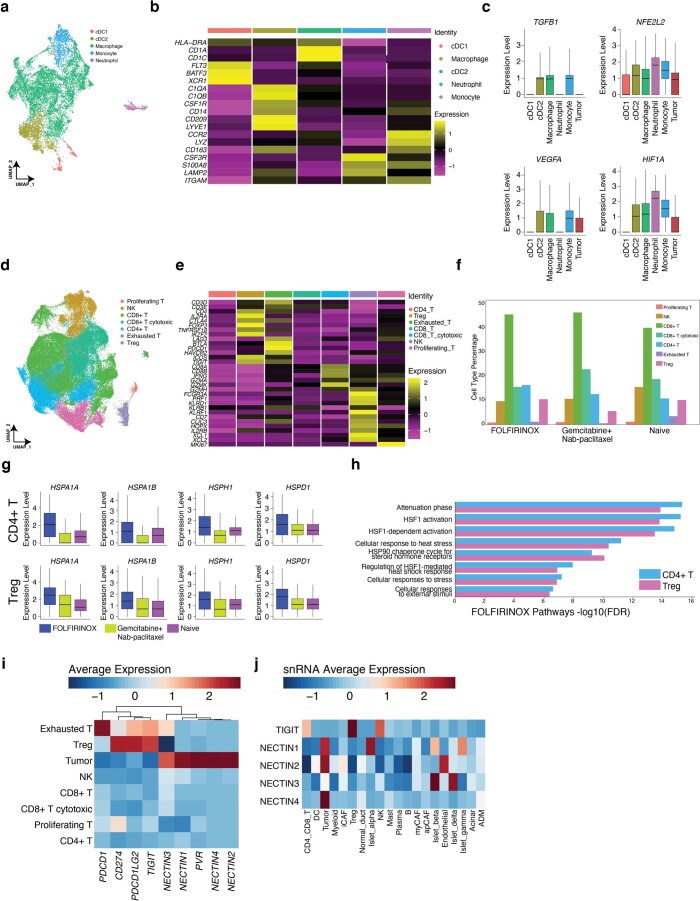
Immune Cells in PDAC. **a)** UMAP of myeloid and dendritic cells (DC) labeled by cell type. **b)** Myeloid and DC cell type marker expression. **c)** Expression of the Keap1-Nrf2 (NFE2L2) pathways genes in all myeloid, DC, and tumor cells in the study. The boxplots show the median with 1.5x IQR whiskers. **d)** UMAP of lymphocyte and NK cells labeled by cell type. **e)** Lymphocyte cell type marker expression. **f)** Cell type percentages of lymphocytes across treatment groups. **g)** Expression of heat shock genes across treatment groups in Treg and CD4 + T cells (cells from n = 26 FOLFIRINOX samples, n = 15 Gemcitabine + Nab-paclitaxel samples, n = 25 untreated samples). The boxplots show the median with 1.5x IQR whiskers. **h)** Pathway enrichment of FOLFIRINOX vs treatment-naïve samples in Treg and CD4 + T cells using gene set overrepresentation analysis. **i)** Average expression of genes in lymphocytes and tumor cells in the scRNA data. **j)** Average expression of *TIGIT* and nectin genes across cell types in the snRNA data.

To examine the immunosuppressed TME characteristics of PDAC^18^, we identified and reclustered immune cells into lymphocytes or myeloid/dendritic cells. In the latter group, we further distinguished between type I and II classical dendritic cells (cDC1, cDC2), macrophages, monocytes and neutrophils. Myeloid cells and classical dendritic cells strongly express TME-remodeling pathway genes, such as angiogenesis and hypoxia pathways, at higher levels than tumor cells (Extended Data Fig. 8a–c). While tumor cells do not have high expression of *NFE2L2* relative to myeloid cells, elevated expression occurs downstream of the Nrf2 pathway (*NQO1*, *GPX2*), which regulates oxidative damage repair (Fig. 8a). Such activation may be triggered via paracrine interactions with TME cells and would indicate that myeloid and dendritic cells contribute towards a pro-tumor TME. Within lymphocytes, we observed slight enrichment of CD4/CD8^+^ T cell subsets in treated samples and expression of heat shock genes in FOLFIRINOX samples (Extended Data Fig. 8d,e,h and Supplementary Note).

We analyzed the expression of common immune checkpoint receptors and ligands, including PD-1 (*PDCD1*), *CTLA4* and *TIGIT* (Fig. 8b). We observed strong expression of exhaustion markers in NK, CD4+ and CD8^+^ T, and regulatory T (Treg) cells. We do not detect any significant expression of immune checkpoint genes in tumor cells or transitioning populations, including *PD-L1* and *PD-L2*, consistent with the poor response of PDAC to anti-*PD-1/PD-L1* immunotherapy^74,75^. Receptor–ligand analyses reveal interactions between the *TIGIT* receptor in lymphocytes and NECTIN ligands across all samples, which we found highly expressed in tumor cells, but somewhat less in other transitioning ductal populations (Fig. 8c and Methods). This is consistent with reports that *NECTIN4* is a potential target for immune checkpoint blockade^76,77^. *TIGIT* interaction with NECTIN inactivates T and NK effector function, which the tumor could exploit for immune evasion^76,77^. *NECTIN1* and *NECTIN4* are most strongly expressed in tumor cells, but *NECTIN2* and *NECTIN3* are also expressed in some lymphocyte cell types, while *TIGIT* is largely expressed in Tregs and exhausted CD4^+^ T cells (Fig. 8c and Extended Data Fig. 8i).

*NECTIN1/2/3* are expressed in fibroblasts, endothelial cells and lymphocytes, respectively, while *NECTIN4* is the most tumor cell-specific NECTIN (Fig. 8c), consistent with previous reports^76^. We analyzed TIGIT and NECTIN expression of individual samples and observed high expression of all NECTIN receptors in tumor cells and *TIGIT* in Tregs and exhausted T cells, and noted substantial heterogeneity across cases, particularly in *TIGIT* expression in exhausted T cells and in *NECTIN1* and *NECTIN3* expression in tumor cells (Fig. 8d). Finally, in the snRNA-seq cohort, we observe the same expression pattern of *TIGIT* and *NECTIN* across cell types (Extended Data Fig. 8j). Using spatial transcriptomics data, we focused on two regions in HT259P1 and HT264P1 to show the expression of *TIGIT* in spots proximal to the infiltrating lymphocyte regions (Fig. 8e). We find colocalization of tumor regions in the H&E with expression of *NECTIN4* across most H&E slides, regardless of treatment status (Fig. 8f). These results provide a rationale for targeting the TIGIT–NECTIN axis to improve anti-tumor T cell activity.

## Discussion

Using bulk sequencing and proteomics/phosphoproteomics, single-cell sequencing, spatial transcriptomics and high-resolution cellular imaging on 83 PDAC samples, we identified transitional populations, including ADM and PanIN, and populations of nontransformed acinar and duct cells and PDAC cells. ADM populations express both oncogenes and tumor suppressor genes, significantly upregulating epithelial-to-mesenchymal transition and stem cell genes, compared with tumor cells^78^. The unique expression pattern of ADM as an intermediate state suggests a dynamic transition between tumor and acinar fates and progression towards PDAC via acquisition of a driver *KRAS* event. This is consistent with acinar sensitivity to *KRAS* mutations as a catalyst of ADM and inclination toward PDAC^78^. Driver mutations mapped to PanIN-like cells and tumor cells, consistent with their role as a precursor lesion. We used pathology-assisted spatial transcriptomics to identify distinct tumor subpopulations, as well as PanIN and PDAC-associated chronic pancreatitis. PanIN profiling by spatial transcriptomics provides direct confirmation of scRNA-seq findings.

CAFs are poorly understood in PDAC. Historically presumed to be cancer drivers, they are now known to have dual behavior as either drivers or suppressors of cancer, depending upon numerous factors^79,80^. We identified iCAFs, myCAFs and apCAFs, further classifying two iCAF subsets as CD133^+^ and CXCR4^+^. We noted that several markers and activated pathways found to be differentially expressed between subtypes are being explored in current clinical trials^15^. We observed higher iCAF abundance in treated samples. This is important, as IL-1-mediated and JAK-STAT signaling in iCAFs have motivated trials of adding IL-1R blockade to standard-of-care (FOLFIRINOX-based) chemotherapy^72^ (ClinicalTrials.gov: NCT02021422) and treating KPC mouse models with a JAK inhibitor decreases tumor size^81^. In patients treated with gemcitabine and nab-paclitaxel (either alone or in succession with other therapies), we observed upregulation of metallothionein genes in iCAFs. Metallothionein proteins are associated with resistance to a variety of chemotherapeutics, and may signal a chemoresistance mechanism^82^.

Immunotherapy has revolutionized treatment of many tumors, but is not yet effective for PDAC^83–85^. Lack of immune checkpoint blockade activity in PDAC is multifaceted, including the shortage of naturally occurring T cell responses, partially due to the inhibition of effective T cell priming and/or T cell exclusion^86,87^. Single-cell analysis revealed that NECTIN family members, especially *NECTIN4*, are tumor-specific, uncovering a potentially targetable interaction with TIGIT in Tregs and exhausted T cells. We observed high expression of all NECTIN genes in tumor cells and *TIGIT* in Tregs and exhausted T cells, but noted substantial heterogeneity across cases. Clarifying the key elements of the immunosuppressive PDAC microenvironment may pave the way for effective immunotherapy in PDAC.

Our study provides a comprehensive analysis of PDAC spatial heterogeneity and treatment effects. We found substantial heterogeneity in PDAC, including spatially separated driver clones, subtype heterogeneity within the same patients and multiple transitional cell populations, including duct-like, ADM and PanIN. Our work provides a resource to identify new targets of clinical relevance. We acknowledge the heterogeneous treatments in the patient population included in the study. Future work using clinical trials, specimens with uniform treatment regimens and comprehensive clinical response data will identify treatment-associated resistance signatures.

## Methods

### Specimens and clinical data

All samples were collected with informed consent in concordance with the Washington University Institutional Review Board (IRB) at the Washington University School of Medicine in St Louis (St Louis, MO). Primary pancreatic adenocarcinoma samples were collected during surgical resection and verified by standard pathology (IRB protocol 201108117). Blood was collected at the time of surgery into vacuum tubes containing heparin or EDTA (BD Bioscience). Cells were isolated by Ficoll-density centrifugation and frozen in FBS with 5% dimethyl sulfoxide. Clinical data were captured in accordance with IRB protocol 20108117, at the time of informed consent, and entered into the REDCap database.

### Sample processing

After verification by an attending pathologist, a 1.5 × 1.5 × 0.5-cm^3^ portion of the tumor was removed, photographed, weighed and measured. Each piece was then subdivided into 6–9 pieces (depending on the original size) and then further subdivided into four transverse cut pieces. Pieces were each then separately placed into formalin, snap-frozen in liquid nitrogen, DMEM and formalin, respectively. The purpose of the switch from punch sampling to the grid processing method was utility-based, as it minimized remainder tissue.

### Genomic DNA and RNA extraction

Tumor tissues and corresponding normal mucosae were obtained from surgically resected specimens, and after a piece was removed for fresh single-cell prep the remaining sample was snap-frozen in liquid nitrogen and stored at −80 °C. Before bulk RNA/DNA extraction, samples were cryo-pulverized (Covaris) and aliquoted for bulk extraction methods. Genomic DNA was extracted from tissue samples with either the DNeasy Blood and Tissue Kit (Qiagen, 69504) or the QIAamp DNA Mini Kit (Qiagen, 51304). Total RNA was extracted with TRI reagent (Millipore Sigma, T9424) and treated with DNase I (Qiagen, 79254) using an RNeasy MinElute Cleanup Kit (Qiagen, 74204). RNA integrity was evaluated using either a Bioanalyzer (Agilent Technologies) or TapeStation (Agilent Technologies). Genomic germline DNA was purified from cryopreserved peripheral blood mononuclear cells using the QiaAMP DNA Mini Kit (Qiagen, 51304) according to the manufacturer’s instructions (Qiagen). The DNA quantity was assessed by fluorometry using the Qubit dsDNA HS Assay (Q32854) according to manufacturer’s instructions (Thermo Fisher Scientific).

### WES

First, 100–250 ng of genomic DNA was fragmented on the Covaris LE220 instrument targeting 250-base pair (bp) inserts. Automated dual-indexed libraries were constructed with the KAPA Hyper library prep kit (Roche) on the SciClone NGS platform (Perkin Elmer). Up to ten libraries were pooled at an equimolar ratio by mass before the hybrid capture targeting a 5-µg library pool. The library pools were hybridized with the xGen Exome Research Panel v1.0 reagent (IDT Technologies) that spans a 39-megabase (Mb) target region (19,396 genes) of the human genome. The libraries were hybridized for 16–18 h at 65 °C followed by stringent washing to remove spuriously hybridized library fragments. Enriched library fragments were eluted and PCR cycle optimization was performed to prevent over amplification. The enriched libraries were amplified with KAPA HiFi master mix (Roche) before sequencing. The concentration of each captured library pool was accurately determined through quantitative PCR (qPCR) utilizing the KAPA Library Quantification Kit according to the manufacturer’s protocol (Roche) to produce cluster counts appropriate for the Illumina NovaSeq-6000 instrument. Then, 2 × 150 paired-end reads were generated targeting 12 gigabases of sequence to achieve ~100x coverage per library.

### RNA-seq

Total RNA integrity was determined using Agilent Bioanalyzer or 4200 Tapestation. Library preparation was performed with 500 ng to 1 μg of total RNA. Ribosomal RNA was blocked using FastSelect reagents (Qiagen) during complementary DNA synthesis. RNA was fragmented in reverse transcriptase buffer with FastSelect reagent and heated to 94 °C for 5 min, 75 °C for 2 min, 70 °C for 2 min, 65 °C for 2 min, 60 °C for 2 min, 55 °C for 2 min, 37 °C for 5 min and 25 °C for 5 min. mRNA was reverse transcribed to yield cDNA using SuperScript III RT enzyme (Life Technologies, per manufacturer’s instructions) and random hexamers. A second strand reaction was performed to yield double-stranded cDNA (ds-cDNA). cDNA was blunt ended, had an A base added to the 3′ ends and then had Illumina sequencing adapters ligated to the ends. Ligated fragments were then amplified for 15 cycles using primers incorporating unique dual index tags. Fragments were sequenced on an Illumina NovaSeq-6000 S4 instrument, generating approximately 30 million paired-end 2 × 150 reads per library.

### Single-cell suspension preparation

For each tumor, approximately 15–100 mg of 2–4 sections of each tumor and/or normal piece of tissue were cut into small pieces using a blade and processed separately. Enzymes and reagents from the Human Tumor Dissociation Kit (Miltenyi Biotec, 130-095-929) were added to the tumor tissue along with 1.75 ml of DMEM. The resulting suspension was loaded into a gentleMACS C-tube (Miltenyi Biotec, 130-093-237) and subjected to the gentleMACS Octo Dissociator with Heaters (Miltenyi Biotec, 130-096-427). After 30–60 min on the heated dissociation program (37h_TDK_1), samples were removed from the dissociator and filtered through a 40-μm Mini-Strainer (PluriSelect no. 43-10040-60) or 40-μm Nylon mesh (Fisher Scientific, 22-363-547) into a 15-ml conical tube on ice. The sample was then spun down at 400*g* for 5 min at 4 °C. After removing the supernatant, when a red pellet was visible, the cell pellet was resuspended using 200 μl to 3 ml of ACK Lysis Solution (Thermo Fisher, A1049201) for 1–5 min. To quench the reaction, 10 ml of PBS (Corning, 21-040-CM) with 0.5% BSA (Miltenyi Biotec, 130-091-376) was added and spun down at 400*g* for 5 min at 4 °C. After removing supernatant, the cells were resuspended in 1 ml of PBS with 0.5% BSA, and live and dead cells were visualized using Trypan Blue. If over 40% of dead cells were present, the sample was spun down at 400*g* for 5 min at 4 °C and subjected to the dead cell removal kit (Miltenyi Biotec, 130-090-101). Finally, the sample was spun down at 400*g* for 5 min at 4 °C and resuspended in 500 μl to 1 ml of PBS with 0.5% BSA to a final concentration of 700 to 1,500 cells per μl.

### Single-nuclei suspension preparation

First, 15–25 mg of pulverized tissue was placed in a 5-ml Eppendorf tube on ice. Using a wide-bore pipette tip (Rainin), a lysis buffer prepared from the Nuclei Isolation protocol (10x Genomics) and SuperRNase inhibitor (Invitrogen) was added to the tube. The tissue solution was gently pipetted until the lysis liquid turned a slightly cloudy color. (The number of pipetting iterations depended on the specific tissue.) The tissue homogenate was then filtered through a 40-μm strainer (pluriSelect) and washed with a BSA wash buffer (2% BSA + 1 × PBS + RNase inhibitor). The filtrate was collected, centrifuged at 500*g* for 6 min at 4 °C and resuspended with a BSA wash buffer. Then, 100 μl of cell lysis solution was set aside for unstained reference, while the rest was stained with 1 μl of 7AAD per 500 μl of the sample. Nuclei underwent FACS and sorting gates were based on size, granularity and dye staining signal. The final suspension was spun down at 500*g* for 6 min at 4 °C, and resuspended with a BSA wash buffer.

### Single-cell/nuclei library prep and sequencing

Utilizing the Chromium Next GEM Single Cell 3′ GEM, Library & Gel Bead Kit v.3.1 and Chromium instrument, approximately 17,500 to 25,000 cells were partitioned into nanoliter droplets to achieve single-cell resolution for a maximum of 10,000 to 15,000 individual cells per sample (10x Genomics, 1000269). The resulting cDNA was tagged with a common 16-nucleotide (nt) cell barcode and 10-nt Unique Molecular Identifier during the reverse transcriptase (RT) reaction. Full-length cDNA from poly-A mRNA transcripts was enzymatically fragmented and size-selected to optimize the cDNA amplicon size (approximately 400 bp) for library construction (10x Genomics). The concentration of the 10x single-cell library was accurately determined through qPCR (Kapa Biosystems) to produce cluster counts appropriate for the HiSeq 4000 or NovaSeq-6000 platform (Illumina). Then, 26 × 98-bp sequence data were generated targeting 50,000 read pairs per cell, which provided digital gene expression profiles for each individual cell.

### Spatial transcriptomics prep and sequencing

Optimal cutting temperature (OCT)-embedded tissues were cryosectioned and placed on a Visium Spatial Gene Expression Slide following Visium Spatial Protocols-Tissue Preparation Guide (10x Genomics, CG000240 Rev A). Briefly, fresh tissues were coated carefully and thoroughly with room temperature OCT without any bubbles. OCT-coated tissues were then placed on a metal block chilled in dry ice until the OCT turned solidified and white. After RNA quality check using Tapestation and morphology check using H&E staining for the OCT-embedded tissues, blocks were scored into a proper size that fit the Capture Areas and then sectioned into 10-μm sections. After the tissue placement into the Capture Area, sections were fixed in methanol, stained with H&E and imaged at ×20 magnification using the brightfield imaging setting on a Leica DMi8 microscope. Tissues were then permeabilized for 18 min and Spatial Transcriptomics libraries were constructed following Visium Spatial Gene Expression Reagent Kits User Guide CG000239 Rev A (10x Genomics). Briefly, cDNA was reverse transcribed from the poly-adenylated messenger RNA which was captured by the primers on the slides. Next, the second strand was synthesized and denatured from the first strand. Free cDNA was then transferred from slides to tubes for further amplification and library construction. Libraries were sequenced on the S4 flow cell of the Illumina NovaSeq-6000 system.

### KPC-OG GEMM mouse model

Three KPC-OG GEMM mice were killed at 3–5 months old, at a time when pathologically these mice have early metaplasia and PanIN throughout the pancreas, with only microscopic PDAC detectable^66,88^. Age-matched KPC-OG negative littermates (CRE and OG negative) were treated with caerulin to induce acute pancreatitis by administering 6 hourly intraperitoneal injections (that is, once per hour for 6 h) at a dose of 100 μg kg^−1^ given every other day for 1 week. For normal, we extracted tissue from KPC-OG breeders negative for cre that underwent no treatment. Cell types were annotated from previous publications^14,89^. All mice were bred and maintained under specific pathogen-free conditions, 12-h light/dark cycle, in accordance with the National Institute of Health and American Association for Accreditation of Laboratory Animal Care (NIH-AALAC) standards and consistent with Washington University School of Medicine Institutional Animal Care and Use Committee (IACUC) regulations (protocol no. 19-0856). Ethical approval for all mouse work was given by Washington University School of Medicine IACUC under protocol no. 19-0856.

### Somatic variant calling

Somatic variants were called from whole-exome tumor-normal paired BAMs using somaticwrapper v.1.5, a pipeline designed for detection of somatic variants from tumor and normal WES data. The pipeline merges and filters variant calls from four callers: Strelka v.2.9.2 (ref. ^90^), VarScan v.2.3.8 (ref. ^91^), Pindel v.0.2.5 (ref. ^92^) and MuTect v.1.1.7 (ref. ^93^). SNV calls were obtained from Strelka, Varscan and MuTect. Indel calls were obtained from Strelka, Varscan and Pindel. The following filters were applied to obtain variant calls of high confidence: normal VAF ≤ 0.02 and tumor VAF ≥ 0.05, read depth in tumor ≥14 and normal ≥8, indel length <100 bp, all variants must be called by 2 or more callers, all variants must be exonic and variants in dbSNP but not in COSMIC excluded.

### KRAS hotspot and within-case genotyping

To verify manually and/or determine the KRAS mutation status at KRAS hotspots G12, G13 and Q61, we used bam-readcount. For each case, we first applied bam-readcount to generate readcounts for each of the nine bases in these loci and then calculated VAF values of all the KRAS hotspots based on reference and alternative base read counts at each position. Additionally, we manually verified every variant present in a sample in a pairwise fashion against other samples within the same case.

### Germline variant calling and annotation

Germline variant calling was performed using an in-house pipeline, germlinewrapper v.1.1 (https://github.com/ding-lab/germlinewrapper), which implements multiple tools for the detection of germline INDELs and SNVs. Germline SNVs were identified using VarScan v.2.3.8 (with parameters: --min-var-freq 0.10 --p-value 0.10 --min-coverage 3 --strand-filter 1) operating on an mpileup stream produced by samtools v.1.2 (with parameters: -q 1 -Q 13) and GATK v.4.0.0.0 (ref. ^94^) using its haplotype caller in single-sample mode with duplicate and unmapped reads removed and retaining calls with a minimum quality threshold of 10. All resulting variants were limited to the coding regions of the full-length transcripts obtained from Ensembl release 95 plus an additional 2 bp flanking each exon to cover splice donor/acceptor sites. We required variants to have allelic depth ≥ 5 reads and alternative allele frequencies ≥ 20% in both the tumor and normal samples. We used bam-readcount v.0.8 for reference and alternative alleles quantification (with parameters: -q 10 -b 15) in both normal and tumor samples. Additionally, we filtered all variants with ≥0.05% frequency in gnomAD v.2.1 (ref. ^95^) and the 1000 Genomes Project^96^.

### Germline variant pathogenic classification

For annotation and prioritization of the filtered germline variants, we used our automatic variant classification tool CharGer v.0.5.4 (ref. ^37^), which computes a classification score based on American College of Medical Genetics and Genomics and the Association for Molecular Pathology (ACMG-AMP) guidelines. CharGer automatically marks as pathogenic those input variants that are marked as known pathogenic in ClinVar’s curated database and marks as likely pathogenic those variants with a CharGer score > 8. All pathogenic or likely pathogenic variants had both their normal and tumor samples reviewed manually by us using the Integrative Genomics Viewer software.

### sc/snRNA-seq data preprocessing

For each sample, we obtained the unfiltered feature-barcode matrix per sample by passing the demultiplexed FASTQs to Cell Ranger v.3.1.0 ‘count’ command using default parameters and the prebuilt GRCh38 genome reference v.3.0.0 (GRCh38 and Ensembl 93) for scRNA or the pre-mRNA version for snRNA. Seurat v.3.1.2 (refs. ^97,98^) was used for all subsequent analyses. First, a series of quality filters was applied to the data to remove those barcodes that fell into any one of these categories recommended by Seurat: too few total transcript counts (<300); possible debris with too few genes expressed (<200) and too few unique molecular identifiers (UMIs) (<1,000); possible more than one cell with too many genes expressed (>10,000) and too many unique molecular identifiers (>10,000); possible dead cell or a sign of cellular stress and apoptosis with too high proportion of mitochondrial gene expression over the total transcript counts (>10%). We constructed a Seurat object using the unfiltered feature-barcode matrix for each sample. Each sample was scaled and normalized using Seurat’s ‘SCTransform’ function to correct for batch effects (with parameters: vars.to.regress = c(‘nCount_RNA’, ‘percent.mito’), variable.features *n* = 2,000). Any merged analysis or subsequent subsetting of cells/samples underwent the same scaling and normalization method. Cells were clustered using the original Louvain algorithm^99^ and top 30 PCA dimensions via ‘FindNeighbors’ and ‘FindClusters’ (with parameters: resolution = 0.5) functions. The resulting merged and normalized matrix was used for the subsequent analysis. Mouse data were aligned to refdata-gex-mm10-2020-A and GFP was added to the reference using the cellranger mkref function (https://support.10xgenomics.com/single-cell-gene-expression/software/pipelines/latest/using/tutorial_mr).

### sc/snRNA-seq cell-type annotation

Main cell types were assigned to each cluster by manually reviewing the expression of a comprehensive set of marker genes (Supplementary Note). These assignments were all done by one person to maximize consistency.

### Spatially distinct tumor cluster assignment

We used sample provenance of tumor cells as well as a requirement of 95% of cells in a tumor cluster to originate from a sample that is physically 6 mm from another sample to conclude that a subcluster is spatially distinct between samples (Supplementary Table 3).

### scVarScan mutation mapping

We applied our in-house tool scVarScan that can identify reads supporting the reference and variant alleles covering the variant site in each individual cell by tracing cell and molecular barcode information in an scRNA bam file. For mapping, we used high-confidence somatic mutations from WES data. Additionally, we use cancerhotspots.org^100^ to obtain the most common KRAS hotspot mutations at G12, G13 and Q61, and use scVarScan to detect potential minority KRAS mutations in each sample.

### scVarScan statistics

To assess the degree of certainty that mutations were preferentially mapped to tumor cells versus nontumor cells (for which mappings can be reasonably assumed to be noise), we devised the following analysis based on the standard binomial difference of proportions test. Let *X*_T_ be the read count for mapped tumor mutations and let *N*_T_ be the total read count (mutation plus reference) for the tumor. Similarly, let *X*_N_ and *N*_N_ be the respective counts for the normal sample. The respective proportions of mapped reads for tumor and normal are clearly *P*_T_ = *X*_T_*/N*_T_ and *P*_N_ = *X*_N_*/N*_N_. Also, define the average joint fraction as *P*_avg_ = *(X*_T_ + *X*_N_*)/(N*_T_ + *N*_N_*)* and its complement as *Q*_avg_ = 1 − *P*_avg_. The large counts we are working with suggest the binomial distribution is well-approximated by the normal (Gaussian) distribution, as assessed by traditional heuristics *N*_T_ *P*_avg_ *Q*_avg_ ≥ 5 and *N*_N_ *P*_avg_ *Q*_avg_ ≥ 5. Adding the standard continuity correction (the normal distribution is continuous, whereas the binomial is discrete), we can then construct the following *Z* score for the difference of proportions:

$$Z_{\mathrm{score}} = \frac{{\left| {P_{\mathrm{T}} - P_{\mathrm{N}}} \right| - \left( {1/N_{\mathrm{T}} + 1/N_{\mathrm{N}}} \right)/2}}{{P_{{\mathrm{avg}}}Q_{{\mathrm{avg}}}\sqrt {1/N_{\mathrm{T}} + 1/N_{\mathrm{N}}} }}\quad \quad \quad \quad \quad \,P = \varPhi \left( {Z_{{\mathrm{score}}}} \right),$$

which is normally distributed with mean 0 and variance 1. The *P* value for the one-sided test of whether the tumor proportion is statistically greater than the normal proportion is *Φ*(*Z*_score_), that is, the area under the standard Gaussian curve within the range *Z*_score_ ≤ *Z* < ∞. We restrict performance of the test only to those cases where *P*_T_ > *P*_N_ is actually observed, skipping cases of *P*_T_ ≤ *P*_N_, to avoid over-correcting in the calculation of false discovery rate (FDR). Using this method, we determined that the rate of mutations mapping is significant in the following comparison: tumor cells versus nontumor cells (*P* ≈ 0), PanIN cells versus nontumor cells (*P* ≈ 0), tumor cells versus PanIN cells (*P* = 1.04 × 10^−11^).

### Single-cell RNA CNV detection

To detect large-scale chromosomal CNVs using single-cell RNA-seq data, inferCNV (v.0.8.2) was used with default parameters recommended for 10x Genomics data. All cells that are not tumor cells were pooled together for the reference normal set. InferCNV was run at a sample level and only with post-quality controlled filtered data. To calculate arm-level CNV events, we used an in-house script to match the gene-level inferCNV output to chromosome bands and take the mean value for each arm.

### Single-cell mutation and CNV plotting

For clarity, we assigned each cell, represented by a single dot in a UMAP plot, with only one genetic alteration, in a hierarchical fashion. For clarity and to not overcomplicate plotting due to too many comparison groups, if a mutation and a copy number event are detected in the same cell, the cell is labeled with the mutation. Additionally, when multiple mutations or copy number events are detected in the same cell, we plot them hierarchically as follows: *KRAS* > *CDKN2A* > *SMAD4* > *TP53*.

### Differential sc/snRNA expression analyses

For cell-level and cluster-level differential expression, we used the ‘FindMarkers’ or ‘FindAllMarkers’ Seurat function as appropriate, using a minimum percentage of 0.25 (parameter min.pct = 0.25) and looking only in the positive direction, as lack of expression is harder to interpret due to the sparsity of the data. The resulting DEGs were then filtered for adjusted *P* < 0.05 and sorted by fold change. All differential expression analyses were carried out using the ‘SCT’ assay.

### Tumor subcluster pathway analysis

To demonstrate tumor heterogeneity in merged scRNA/snRNA data, we first took subsets of tumor cells from each individual case and renormalized with Seurat function ‘SCTransform’. We then found case-level clusters with Seurat functions ‘FindNeighbors’ and ‘FindClusters’ (top 20 PCA dimensions, resolution = 0.8). Clusters with fewer than 0.1% of total tumor cells across cases were excluded. For each case-level cluster, we found DEGs with function ‘FindAllMarkers’ with a minimum percentage (min.pct) of 0.1, a minimum percentage difference (min.diff.pct) of 0.1, positive log fold change and adjusted *P* < 0.05. For each DEG list, we ran an enrichment analysis using the function ‘enricher’ against the 50 MSigDB hallmark gene sets^32,101^. The universe background for enrichment analysis was composed of genes detected in more than 0.1% of total tumor cells across cases. For each pathway, genes shown as enriched with adjusted *P* < 0.05 in any cluster were used to calculate the pathway score. Finally, in the merged tumor cell object, we calculated the average expression of genes identified in each pathway, centered and scaled across all clusters as the final score. To present pathways that distinguish tumor clusters the most, we ranked tumor cell-related pathways by their occurrence shown as enriched significantly (adjusted *P* < 0.05) in the enrichment analysis and plotted the most common pathways. The final heatmap was generated with the pheatmap package using the Optimal Leaf Ordering clustering method from the seriation package.

### Receptor–ligand interactions

We used the CellPhoneDB tool^102^ to detect significant pairs of receptor–ligand interactions between cell types. This comparison was done at the sample level using default parameters between tumor and lymphocyte cell types.

### Monocle trajectory analysis

We used the Monocle3 tool (https://cole-trapnell-lab.github.io/monocle3/) to infer cell-type transition states among acinar, transitional, PanIN and normal ductal populations. Objects and trajectory mapping were obtained by following tutorials outlined by developers (https://cole-trapnell-lab.github.io/monocle3/).

### CopyKAT

To predict copy number ploidy without tumor annotation we utilized CopyKAT (https://github.com/navinlabcode/copykat) and followed the standard tutorial to define populations of aneuploid tumor cells.

### Spatial transcriptomics data preprocessing

For each sample, we obtained the unfiltered feature-barcode matrix per sample by passing the demultiplexed FASTQs and associated H&E image to Space Ranger v.1.1.0 ‘count’ command using default parameters with reorient-images enabled, and the prebuilt GRCh38 genome reference 2020-A (GRCh38 and Ensembl 98). Seurat v.4.0.3 was used for all subsequent analyses. We constructed a Seurat object using the ‘Load10X_Spatial’ function for every slide. Each slide was then scaled and normalized with the ‘SCTransform’ function to correct for batch effects (with parameters: vars.to.regress = c(‘nCount_Spatial’)). Any merged analysis or subsequent subsetting of cells/samples for a sample with several slides underwent the same scaling and normalization method. Spots were clustered using the original Louvain algorithm^99^ and top 20 PCA dimensions via ‘FindNeighbors’ and ‘FindClusters’ functions.

### sc/snRNA-seq cell-type annotation

For spot-level cell-type assignment, we used the Seurat functions ‘FindTransferAnchors’ and ‘TransferData’ to perform a cell-type label transfer from the paired snRNA-seq annotations to the spatial transcriptomics spots. For further resolution, we used RCTD to deconvolve cell types within a given spot^35^. We used the default parameters in RCTD using the ‘multi’ mode and a minimum of 25 nuclei for each cell-type identity to deconvolve; https://github.com/dmcable/spacexr.

### Manual spot selection

In select samples with PanIN identified and a tumor or normal ductal structure within the capture area, we annotated the spatial transcriptomics spots using the Loupe Browser 5.0 and the lasso tool to manually select and annotate groups of spots. Annotated spots were then used to annotate the UMAP object; then, annotated spots were subsetted from the full object and DEGs were calculated using Seurat (FindAllMarkers).

### DNA and RNA sample quality control

Bulk sequencing data quality metrics (adaptor content, mapping quality, coverage and swaps/mislabeling) were determined for DNA and RNA bams using our in-house pipeline SeqQEst. The inclusion criteria for paired DNA and RNA bams with sufficient coverage was >50× coding region coverage in WES or >50 Mb mapped depth in RNA-seq data.

### RNA quantification

We used our in-house bulk RNA-seq expression analysis pipeline for quantification. Briefly, for each sample, the raw sequence reads were aligned into BAM files using STAR^103^ (v.2.7.4a) two-pass alignment with GRCh38 as the reference. The resulting BAM files were then quantified as a raw count-matrix using read feature counts using Subread^104^ (v.2.0.1). For both alignment and quantification, gene annotations were based on Gencode v.34. The raw counts were then converted to FPKM-UQ based on GDC’s formula (https://docs.gdc.cancer.gov/Data/Bioinformatics_Pipelines/Expression_mRNA_Pipeline/#upper-quartile-fpkm) and then log_2_ transformed with 1 pseudocount.

### Proteomic and phosphoproteomics quantification

Proteomic data processing followed the methods detailed by Clark et al.^105^. Briefly, raw mass spectrometry files were converted into open mzML format, then searched using the MSFragger database against a RefSeq protein sequence database appended with an equal number of decoy sequences. The specific parameters and software are detailed in the Clark et al. 2020 study. We then used the ComBat function from the R sva package to correct for TMT batch effects^106^.

### Pathway analysis

For each comparison, we obtained the top 30 genes ranked by highest fold change that are significantly different between the comparison groups (FDR < 0.05). We used ConsensusPathDB-human for gene set over-representation analysis^107^.

### Statistics and reproducibility

Relevant statistics are referred to in each of the associated methods sections. We did not use statistical methods to predetermine a sample size and patients were not randomly selected, as they were enrolled as they passed through the clinic. We excluded samples that did not pass sample prep quality control. For all immunofluorescence imaging, at least three regions of each sample were assayed, but immunofluorescence staining was not repeated for the same sample sections.

### Reporting summary

Further information on research design is available in the Nature Research Reporting Summary linked to this article.

## Online content

Any methods, additional references, Nature Research reporting summaries, source data, extended data, supplementary information, acknowledgements, peer review information; details of author contributions and competing interests; and statements of data and code availability are available at 10.1038/s41588-022-01157-1.

## Supplementary information

Supplementary Information Supplementary Note and Fig. 1.

Reporting Summary

Supplementary Tables Supplementary Tables 1–5.

Supplementary Data 1 Bulk omics data including somatic and germline variants and proteogenomics data.

Supplementary Data 2 CNVkit raw copy number calls across the sample set.

Supplementary Data 3 Total cell count of transitional cell populations and mutation mapping across samples.

Supplementary Data 4 Differentially expressed genes (DEGs) identified by annotating spatial transcriptomics spots using the Loupe Browser and Seurat. FindAllMarkers function from Seurat was used to identify DEGs.

Supplementary Data 5 Tumor purity estimates predicted by ABSOLUTE across sample cohort.

## Data Availability

All human sequencing and imaging data have been deposited via the Human Tumor Atlas Network (HTAN) dbGaP Study Accession: phs002371.v1.p1 (https://www.ncbi.nlm.nih.gov/projects/gap/cgi-bin/study.cgi?study_id=phs002371.v1.p1). In addition, all data have been deposited to the HTAN Data Coordinating Center Data Portal at the National Cancer Institute: https://data.humantumoratlas.org/ (under the HTAN WUSTL Atlas). References (GRCh38 genome reference v3.0.0 and refdata-gex-mm10-2020-A) used for single-cell analysis of the human and mouse genomes, respectively, are available from public sources, as described in access scripts freely furnished by 10x Genomics: https://support.10xgenomics.com/single-cell-gene-expression/software/release-notes/build. Mouse single-cell RNA-seq data are freely available from the National Library of Medicine BioProject (https://www.ncbi.nlm.nih.gov/bioproject/) under accession: PRJNA835747. Data for single-cell integration from Peng et al.^24^ were downloaded from the Genome Sequence Archive (PRJCA001063).

## Code availability

The code for the mutation mapping from bulk to single cells can be found at: https://github.com/ding-lab/10Xmapping. Code for inferCNV post processing can be found at: https://github.com/ding-lab/infer_cnv_postprocesssing.git. Code for germline wrapper variant calling can be found at: https://github.com/ding-lab/germlinewrapper.
