# Supplementary material for: Spatially restricted drivers and transitional cell populations cooperate with the microenvironment in untreated and chemo-resistant pancreatic cancer
Source: Nat Genet. 2022 Aug 22;54(9):1390–405. doi: 10.1038/s41588-022-01157-1 (PMC9470535; doi:10.1038/s41588-022-01157-1)
Supplement: Supplementary file 1 — Supplementary Note and Fig. 1. [file 41588_2022_1157_MOESM1_ESM.pdf]

nature portfolio

https://doi.org/10.1038/s41588-022-01157-1

Supplementary information

Spatially restricted drivers and transitional cell populations cooperate with the microenvironment in untreated and chemo-resistant pancreatic cancer

In the format provided by the authors and unedited

Supplementary Note

Bulk RNA-seq subtype classification and estimation

Moffitt subtyping was consistent within-tumor across samples, while Collisson and Bailey subtyping showed larger numbers of subtype heterogeneity across samples. We observed differences in immune subtypes between spatial samples in 7 tumor cases (**Extended Data Fig. 1g**). We also observed a moderate positive correlation between scRNA stroma percentages and ESTIMATE stroma scores (Spearman R: 0.46, p = 10^{-4}) though not between scRNA immune fraction percentages and ESTIMATE immune scores (**Extended Data Fig. 2a-b**).

Spatial Heterogeneity in Bulk Proteomics

In order to assess inter- and intra-tumor heterogeneity at the bulk proteomics level, we conducted a global pairwise correlation analysis for the n = 30 samples from 9 tumor cases with proteomics data (**Methods, Supplementary Table 4**). While most samples that originate from the same case had strong correlation with each other, we identified three cases (indicated by colored boxes) that showed significantly lower intra-tumor correlation (**Extended Data Fig. 2g,h and Methods**). Using paired scRNA data from the corresponding samples, we evaluated the cell type composition which likely contributes to the heterogeneity observed at the protein level. For instance, in case HT064P1, samples S1H7 and S1H9 are different from samples S1H1 and S1H3 as they have lower tumor content and higher B cell content (**Extended Data Fig. 2i and Supplementary Data Fig. 1a,b,c**).

Additionally, we determined the impact of mutations on downstream targets by analyzing the associated changes at protein and phosphorylation levels in several oncogenic pathways (**Methods**). We observed that samples carrying a *TP53* mutation had several proteins and phosphosites upregulated in the cell cycle and DNA mismatch repair pathways, including MCM7, CDK1, and the proliferation marker MKI67 (**Supplementary Data Fig. 1d,e**). As expected, several members of the RTK Ras pathway had higher phosphorylation in *KRAS* mutants (**Supplementary Data Fig. 1e**). We further analyzed the abundance of key phosphosites in the *KRAS* pathway across spatial samples (**Methods**). Strikingly, we found a large degree of differential regulation, both between and within tumors, in several phosphosites within the PI3K/PDk1/Akt and Raf/Mek/Erk pathways (**Fig. 3e**). There is a modest association between *TP53* mutation status and lower phosphorylation levels in MAPK1 and MAPK3, among others (Wilcoxon rank sum p = 0.028 and p = 0.087, respectively) (**Supplementary Data Fig. 1f,g**). In

HT125P1, two samples (S1H3 and S1H9) do not have a detectable KRAS mutation while the other two (S1H4 and S1H8) have a G12V mutation, and these samples segregate accordingly into the higher and lower phospho-signaling groups, suggestive of differential RAS activation within the same patient. In particular, the MAPK1 T185 phosphosite is differentially regulated between these samples, while MAPK1 Y187 is uniformly expressed throughout HT125P1. Furthermore, the three cases enclosed in colored boxes, which match the heterogeneous cases in **Extended Data Fig. 2h**, show consistent heterogeneous protein signaling in the samples with different cell type composition, pointing to the importance of spatial sampling to obtain the full picture of heterogeneity in PDAC (**Extended Data Fig. 2h,i**).

Gene Expression and CNV Model for HT061P1

The tumor subpopulation in punch A is initially driven by *KRAS* G12V and the *KRAS* G12V population, then acquired an amplification in *GATA6* and a 17p deletion. In punches B and C (and a minute portion of A), the initial driver was *KRAS* G12D, followed by a gain of *AKT2*, *MYC*, and 1q, with additional subsets of cells acquiring either *ERBB2* or *GATA6* amplification and *PTEN* deletion (**Fig. 3d,e and Extended Data Fig. 2f**). Given the extent of heterogeneity observed across all samples, these results suggest a vast degree of tumor heterogeneity in PDAC generally at the expression, mutational, and CNV levels that may impact tumor growth and progression (**Extended Data Fig. 2g**).

CAF TME-Remodeling Pathways

To better understand the role of CAFs in tumorigenesis, we analyzed the expression patterns of CAF subtypes to test whether they were enriched for TME-remodeling pathways. Aside from myCAFs, we observed a significant reduction of *CAV1* and *CAV2* expression, previously linked to poor clinical outcomes[1,2], in all CAFs compared to fibroblasts present in NAT samples (**Extended Data Fig. 7c**). In addition to their very high levels of CXCR4 expression, CXCR4+ iCAFs express high levels of its ligand, *CXCL12* (**Extended Data Fig. 7d**)[3–5]. While apCAFs have high expression of *NFE2L2,* which is involved in oxidative damage repair, myCAFs have high expression of *HIF1A,* which regulates tolerance to hypoxic environments (**Extended Data Fig. 7e**). Together, our results suggest different roles of CAF subtypes in remodeling of the TME[6,7].

Lymphocyte Subsets Breakdown

Within the lymphocyte subsets, we assigned states to CD4+ and CD8+ T based on their exhaustion, proliferation, and cytotoxic markers (**Extended Data Fig. 8d,e**). We observed similar

percentages of cell types across treatment groups, with slightly higher abundance of CD4+ T cells in FOLFIRINOX samples and higher numbers of CD8+ T cells in treated samples (**Extended Data Fig. 8f**). CD4+ T cells and Tregs in FOLFIRINOX samples had high expression of heat shock genes, such as *HSPA1A, HSPA1B, HSPH1,* and *HSPD1,* compared to other treatment groups (**Extended Data Fig. 8g**). Further, pathway enrichment analyses revealed a large number of cellular responses to heat stress in both CD4+ T cells and Tregs in these samples (**Extended Data Fig. 8h**).

Pathological Parameters and Assessment

Each tumor that is subdivided into smaller increments is subjected to H&E staining and assessed by a pathologist for the following parameters: percentage of viable tumor present, tumor differentiation, presence of recognizable pancreatic parenchyma surrounding or interspersed between tumor, lymphovascular invasion, and perineural invasion. Both slices of each tumor piece, both L1 and L4 when available, were subjected to assessment. For the correlation with scRNA-based tumor percentages, we averaged the top and bottom slide (L1 and L4) tumor estimates.

sc/snRNA-seq Cell Type Annotation

Main cell types were assigned using the following marker genes used were *KRT19, KRT8, KRT18, KRT17, KRT7, KRT5, KRT6A, KRT14, EPCAM, TACSTD2, ANXA2, S100A10, S100A11, S100A16, TPM1, TFF1, S100A6, AGR2, C19orf33* (tumor); *INS, GCG, SST, GHR, PPY, GCK, PCSK1, PCSK2, CHGA, CHGB, SYP, KCNJ11* (islet); *CTRB1, CELA3A, CELA3B, CTRB2, PLA2G1B, PRSS2, SPINK1, CLPS, CPA1, PRSS1, CPA2, REG1A, PNLIP, SYCN, PNLIPRP1, CTRC, KLK1, CELA2A, CPB1* (acinar); *VWF, PECAM1, FLT4, FLT1, FLT3, KDR, PLVAP, ANGPT2, TRIM24, ACTA2* (endothelial); *TIMP1, FN1, POSTN, ACTA2, BST2, LY6D, COL6A1, SLC20A1, COL6A2, CD9, S100A4, EMP1, LRRC8A* (fibroblast); *HBD, GYPA, HBA1, HBA2, CA1, HBB, BRSK1* (erythrocyte); *SDC1, IGHG1, IGHG3, IGHG4* (plasma); *CD19, MS4A1, CD79A, CD79B, CD83, CD86* (B cells); *CD8B, CD8A, CD3E, CD3D* (CD8 T cells); *CD8B, CD8A, CD4* (CD4 T cells); *XCL2, XCL1, SPON2, KLRF1, KIR2DL3, IL2RB, HOPX, CLIC3, CD7, KLRB1, KLRD1, GZMA, PRF1, CD160, NCAM1, FCGR3A* (NK); *FCER1A, KIT, FCER2, ENPP3* (mast); *CD1C, CD1A, FLT3, ZBTB46, XCR1, CLEC9A, IRF8, FLT3, ZBTB46, BATF3* (cDCs); *LY6G6D, MPO, FUT4, FCGR3A* (neutrophils); *ITGAM, LGALS3, CD68, CD163, LYZ, ADGRE1, LAMP2* (macrophages); *CD14, FCGR3A, FCGR1A* (monocytes). We further subdivided certain cell types into subtypes or cell states using the following: *IKZF2, TNFRSF18, FOXP3, CTLA4, IL7R, IL2RA*

(Treg); *GZMH, GZMK, GZMB, GZMA, PRF1, IFNG, FASLG, LAMP1, CD8A, CD8B, CD3E, CD3D* (cytotoxic T cell); *VSIR, TIGIT, ICOS, EOMES, HAVCR2, PDCD1, BTLA, CD244, LAG3, CD160, CTLA4, CD96* (exhausted T cell); *MKI67* (proliferation marker); *ACTA2, FAP, PDPN, PDGFRA* (general CAF); *IL6, CXCL1, CXCL12, CXCL2* (iCAF); *ACTA2, THY1, TAGLN* (myCAF); *CD74, SAA2, SAA1* (apCAF). For ADM populations, we used a combination of tumor and acinar markers. For the mouse samples the following marker genes used were *Apoe, Saa3, C1gc* (macrophage); *Ear2, Lyz1* (myeloid cells); *Cd79a, Ly6d, Ms4a1*(b cells); *S100a8, S100a9, G0s2* (neutrophils); *Dcn, Col1a1, Col3a1* (fibroblasts); *Ccl5, Cd3g, Gzma, Nkg7* (t and nk cells); *Clu, Tff1, Krt18, Krt8, Krt19, Krt7, Epcam* (ductal cells); *Igfbp7, Plvap, Cd34* (endothelial cells); *Ccr7, Ccl22* (dendritic cells); *Ctrb1, Prss2, Ty5* (acinar cells); *Rgs5, Acta2* (perivascular); *Cdkn2a, S100a6, Igfbp4, Sparc, Vim, Spp1* (EMT-like); *Gfp* (metaplastic cells: tumor + Acinar_GFP).

Integration of Single-cell Data with Previous Publications

While our study profiled a total of 232,764 cells and 83,860 nuclei, we profiled the presence of the transitional populations in the context of another recent study by Peng et al., who profiled 57,530 cells from PDAC samples and control pancreases[8]. We employed the same filtering strategy discussed in Methods and integrated both datasets using rpca. Using the markers from Peng. et al. (e.g. acinar: PRSS1, CTRB1, CTRB2, and REG1B; ductal: MBP, CFTR, MMP7, KRT19, KRT7, TSPAN8, and SLPI), we identified the immune cell types along with acinar, ductal, and tumor populations and integrated them with our single-cell RNAseq samples.

Protein Extraction and Lys-C/Trypsin Tandem Digestion

Tissue lysis and downstream sample preparation for global proteomic and phosphoproteomic analysis were carried out as previously described[9,10] . Approximately 25–50 mg of each cryo-pulverized HTAN tissue was resuspended in lysis buffer (8 M urea, 75 mM NaCl, 50 mM Tris, pH 8.0, 1 mM EDTA, 2 µg/mL aprotinin, 10 µg/mL leupeptin, 1 mM PMSF, 10 mM NaF, Phosphatase Inhibitor Cocktail 2 and Phosphatase Inhibitor Cocktail 3 [1:100 dilution], and 20 µM PUGNAc) by repeated vortexing. Lysates were clarified by centrifugation at 20,000 g for 10 min at 4°C, and protein concentrations were determined by BCA assay (Pierce). Proteins were reduced with 5 mM dithiothreitol (DTT, ThermoFisher) for 1 h at 37°C, and subsequently alkylated with 10 mM iodoacetamide (Sigma) for 45 min at room temperature (RT) in the dark. Samples were diluted 1:4 with 50 mM Tris-HCl (pH 8.0) and subjected to proteolytic digestion with LysC (Wako Chemicals) at 1 mAU:50 mg enzyme-to-substrate ratio for 2 h at RT, followed by the addition of sequencing grade modified trypsin (Promega) at 1:50 enzyme-to-substrate ratio and overnight

incubation at RT. The digested samples were then acidified with 50% formic acid (FA, Fisher Chemicals) to pH 2. Tryptic peptides were desalted on reversed phase C18 SPE columns (Waters) and dried using Speed-Vac (Thermo Scientific).

TMT-11 Labeling of Peptides

Dried peptides from each sample were labeled with 11-plex TMT (Tandem Mass Tag) reagents (Thermo Fisher Scientific). 200 µg of peptides from each of the HTAN samples was dissolved in 80 µL of 100 mM HEPES, pH 8.5 solution. 30 HTAN samples were labeled in 3 TMT sets. A reference sample was created by pooling an aliquot from 26 HTAN samples (representing ~90% of the sample cohort) and was included in all TMT 11-plex sets as a pooled reference channel (Channel 126). 5 mg of TMT reagent was dissolved in 500 µL of anhydrous acetonitrile, and then 30 µL of each TMT reagent was added to the corresponding aliquot of peptides. After 1 h incubation at RT, the reaction was quenched by incubation with 5% NH_2OH for 15 min at RT. Following labeling, peptides were desalted on reversed phase C18 SPE columns (Waters) and dried using Speed-Vac (Thermo Scientific).

Peptide Fractionation by Basic Reversed-phase Liquid Chromatography (bRPLC)

To reduce the likelihood of peptides co-isolating and co-fragmenting due to high sample complexity, we employed extensive, high-resolution fractionation via basic reversed phase liquid chromatography (bRPLC). For each TMT set, about 2.2 mg of desalted peptides was reconstituted in 900 µL of 5 mM ammonium formate (pH 10) and 2% acetonitrile (ACN) and loaded onto a 4.6 mm x 250 mm RP Zorbax 300 A Extend-C18 column with 3.5-mm size beads (Agilent). Peptides were separated using an Agilent 1200 Series HPLC instrument using basic reversed-phase chromatography with Solvent A (2% ACN, 5 mM ammonium formate, pH 10) and a non-linear gradient of Solvent B (90% ACN, 5 mM ammonium formate, pH 10) at 1 mL/min as follows: 0% Solvent B (7 min), 0% to 16% Solvent B (6 min), 16% to 40% Solvent B 60 min), 40% to 44% Solvent B (4 min), 44% to 60% Solvent B (5 min) and then held at 60% Solvent B (14 min). Collected fractions were concatenated into 24 fractions as described previously[10]; 5% of each of the 24 fractions was aliquoted for global proteomic analysis, dried down in a Speed-Vac, and resuspended in 3% ACN, 0.1% formic acid prior to ESI-LC-MS/MS analysis. The remaining sample was utilized for phosphopeptide enrichment.

Enrichment of Phosphopeptides by Fe-IMAC

The remaining 95% of the fractions were further concatenated into 12 fractions prior to phosphopeptide enrichment using immobilized metal affinity chromatography (IMAC) as

previously described[10]. In brief, Ni-NTA agarose beads were utilized to prepare Fe^{3+}-NTA agarose beads, and then about 200 µg of peptides of each fraction reconstituted in 80% ACN/0.1% trifluoroacetic acid were incubated with 10 µL of the Fe^{3+}-IMAC beads for 30 mins. Samples were then spun down, and the supernatant containing unbound peptides was removed. The beads were brought up in 80% ACN, 0.1% trifluoroacetic acid and then loaded onto equilibrated C-18 Stage Tips, and washed by 80% ACN, 0.1% trifluoroacetic acid, rinsed twice with 1% formic acid, followed by sample elution off the Fe^{3+}-IMAC beads with 100 µL of 500 mM dibasic potassium phosphate, pH 7.0. C-18 Stage Tips were then washed twice with 1% formic acid, followed by elution of the phosphopeptides from the C-18 Stage Tips with 80 µl of 50% ACN, 0.1% formic acid twice. Samples were dried down and resuspended in 3% ACN, 0.1% formic acid prior to ESI-LC-MS/MS analysis.

ESI-LC-MS/MS for Global Proteome and Phosphoproteome Analysis

The global proteome and phosphoproteome fractions were analyzed as described in a previous study[9]. Peptides (~0.8 µg) were separated on an Easy nLC 1200 UHPLC system (Thermo Scientific) on an in-house packed 20 cm x 75 mm diameter C18 column (1.9 mm Reprosil-Pur C18-AQ beads (Dr. Maisch GmbH); Picofrit 10 mm opening (New Objective)). The column was heated to 50°C using a column heater (Phoenix-ST). The flow rate was 0.300 µL/min with 0.1% formic acid and 2% acetonitrile in water (A) and 0.1% formic acid, 90% acetonitrile (B). The global peptides were separated with a 6–30% B gradient in 84 mins and analyzed using the QE-HFX (Thermo Scientific). Parameters were as follows MS1: resolution – 120,000, mass range – 400 to 2000 m/z, RF Lens – 30%, AGC Target 3e6, Max IT – 50 ms, charge state include - 2-5, dynamic exclusion – 20 s, top 20 ions selected for MS2; MS2: resolution – 45,000, collision energy NCE – 32, isolation width (m/z) – 0.7, AGC Target – 1.0e5, Max IT – 96 ms. The phosphopeptides were separated with a 6–30% B gradient in 84 mins and analyzed using the Lumos (Thermo Scientific). Parameters were as follows MS1: resolution – 60,000, mass range – 350 to 1800 m/z, RF Lens – 30%, AGC Target 4.0e5, Max IT – 50 ms, charge state include - 2-6, dynamic exclusion – 45 s, top 20 ions selected for MS2; MS2: resolution – 50,000, HCD collision energy – 34, isolation width (m/z) – 0.7, AGC Target – 2.0e5, Max IT – 100 ms.

CiVIC Drug Matching

We obtained evidence of expression-based response to drugs from CIViC[11]. We filtered the database for only sensitive results and positive direction (i.e., "expression" and "overexpression").

We then matched these annotations to upregulated DEGs in our comparison groups. In the present study we used the 06/02/2020 nightly clinical evidence summary annotations.

Copy Number

The somatic copy number alterations (CNAs) are predicted using CNVkit (v0.9.6)[12]. For paired tumor-normal samples, the matched normal was used as the reference to determine CNAs of the tumor sample. Low quality CNAs were filtered based on the coverage (<20), the number of probes (<10), and length (< 10 kb). To define copy numbers from CNVkit, the thresholds are as follows: -t -1.1, -0.2, 0.2, 0.7. Due to low purity of the sample set, pathologist purity estimates were provided (-U) for copy number calling along with the clonal filter (-m).

ABSOLUTE

Tumor purity estimates were predicted using ABSOLUTE (v1.0.6)[13]. Segment files for ABSOLUTE were generated using cnvkit export seg command. Default parameters were used as defined by run example usage (https://software.broadinstitute.org/cancer/cga/absolute_run) with the following modifications: max.as.seg.count=8000 and platform="Illumina_WES", copy_num_type="total" and primary.disease="Pancreatic Cancer". Purity estimates are included in **Supplementary Data 5**. We compared estimates of WES-based tumor cellularity using ABSOLUTE with scRNA and pathology estimates and obtained similar correlation rates (**Supplementary Data 5, Supplementary Table 5**).

ESTIMATE Immune and Stroma Scores

Scores reflecting the overall immune and stromal infiltration and tumor purity estimation were calculated by the R package ESTIMATE[14] using the normalized RNA expression data (FPKM-UQ). The ESTIMATE algorithm is based on single-sample gene set enrichment analysis and generates three scores: 1) stromal score (which captures the presence of stroma in tumor tissue), 2) immune score (which represents the infiltration of immune cells in tumor tissue), and 3) estimate score (which infers tumor purity).

Immunofluorescence Staining of FFPE Slides

FFPE samples were first cut into 4-μm tissue sections and heated at 55°C for 5 hours prior to staining. Then, deparaffinization and rehydration was performed via incubation subsequently in xylene, 100%, 95%, 70%, 50%, and 25% ethanol. After another 2-minute wash in ddH2O, antigen retrieval was performed in a hot-water bath using a 10mM sodium citrate buffer at 80-90°C for 22

minutes. The slides were then cooled down to room temperature. After that, glycine blocking was then performed using 100mM glycine, followed by two washes in PBST. Sections were then circled with a PAP pen, and blocked in the blocking buffer (10% normal donkey serum in 1% BSA) for one hour. Next, primary antibodies incubated on the tissues at 4°C overnight. The next day, the slides were washed twice for 5 minutes in 1x PBST. Secondary antibodies (Alexa Fluor 488 AffiniPure F(ab')2 Fragment DαRabbit, Alexa Fluor 555 AffiniPure F(ab')2 Fragment DαMouse, Alexa Fluor 647 AffiniPure F(ab')2 Fragment DαRat) were then incubated at room temperature for one hour. Following two more 5-minute washes in PBST, sections were treated with Hoechst stain at a concentration of 1:2000 for 8 minutes. Sections were then washed in 1x PBS twice for 3 minutes, and then mounted in an aqueous mounting medium and covered with a coverslip. Pressure was applied to the sections to ensure total coverage by the mounting medium. Finally, slides were sealed with nailpolish and images were taken in different fluorescence channels. Dilutions for antibodies are as follows: α-Amylase, Sigma, Catalog: A8273 (1:150), Cytokeratin 19, Santa Cruz, Catalog: sc-376126 (1:100), Ki67, Thermo, Catalog: 14-5698-82 (1:50), Alexa Fluor 488 AffiniPure F(ab')2 Fragment DαRabbit, Jackson Immuno Research, Catalog: 711-546-152 (1:1000), Alexa Fluor 555 AffiniPure F(ab')2 Fragment DαMouse, Thermo, Catalog: A31570 (1:1000), Alexa Fluor 647 AffiniPure F(ab')2 Fragment DαRat, Jackson Immuno Research, Catalog: 712-606-153 (1:1000), Hoechst, Life Technologies, Catalog: H3570 (1:2000).

Expression-Based Subtyping

Bulk expression data from HTAN (65 cases) and CPTAC (137 cases) were combined for expression-based subtyping. Briefly, gene signatures detected in both cohorts were selected from each gene marker set. (Bailey N=4414 Moffitt N=50, and Collisson N=60) Combined expression data of log2 upper quartile normalized FPKM reads from both cohorts were first corrected for batch effect using ComBat function in sva package[15], then z-score normalized gene-wise before clustering with the ConsensusClusterPlus (Wilkerson and Hayes, 2010) package in R. (Default parameters. 1000 iterations). The optimum k for each marker set was selected based on the number of subtypes originally identified in each gene marker set (2, 3, and 4 for Moffitt, Collisson, and Bailey, respectively.) The clusters were then assigned to each subtype by inspecting the expression level of their respective marker gene lists.

Immune Clustering Using Cell Type Enrichment Scores

The abundance of each cell type was inferred by the xCell web tool[16], which performed the cell type enrichment analysis from gene expression data for 64 immune and stromal cell types (default

xCell signature). xCell is a gene signatures-based method learned from thousands of pure cell types from various sources. We used the FPKM-UQ expression matrix as the input to xCell. xCell generated an immune score per sample that integrates the enrichment scores of various cell types, including B cells, CD4+ T-cells, CD8+ T-cells, DC, eosinophils, macrophages, monocytes, mast cells, neutrophils, and NK cells. Immune subtypes of HTAN PDAC cohorts were generated based on the consensus clustering of the xCell cell type enrichment scores (Wilkerson and Hayes, 2010). Among the 64 cell types tested in xCell, we selected cell types with at least 2 samples with xCell enrichment $p < 0.01$ and performed the consensus immune clustering based on the z-score normalized xCell enrichment scores. The consensus clustering was determined by the R package ConsensusClusterPlus (parameters: reps = 2000, pItem = 0.9, pFeature = 0.9, clusterAlg = "kmdist", distance = "spearman").

Mutation Impact on the Proteome and Phosphoproteome

We used an aggregated database of interacting proteins that combines Omnipath, DEPOD, CORUM, Signor2, and Reactome databases as previously described[17]. We focused our analyses on PDAC SMGs previously reported in the literature, but only KRAS and TP53 had large enough numbers in each comparison group for sufficient statistical power[18]. For each interacting pair, we split samples with and without mutations in partner A and compared expression levels (both protein and phosphosites) both in *cis* (partner A) and in *trans* (partner B). We calculated the median difference in expression and tested for significance using two-sided Wilcoxon rank sum tests. We further refined the list of *trans* interactions by filtering proteins that are not part of oncogenic processes identified in TCGA[19].

KRAS Phosphosignaling Analysis

Oncogenic KRAS signaling in PDAC is believed to pass through three major pathways: Raf/Mek/Erk, PI3K/Pdk1/Akt, and the Ral guanine nucleotide exchange factor pathway[20]. We focused on the Raf/Mek/Erk pathway (along with PI3K/Pdk1/Akt) because its signaling is controlled by phosphorylation. We used the phosphosites identified in this pathway as detailed in Collisson et al[21].

Differential Proteogenomic Analysis

For differential analysis between groups of samples using bulk data (gene expression, proteomics, and phosphoproteomics), we used two-sided Wilcoxon rank-sum tests to test for differential abundances of genes, proteins, and phosphosites. We required that at least 50% of

samples in each comparison group have non-missing values. P-values were then adjusted using the Benjamini-Hochberg multiple test correction to obtain features with an FDR cutoff ≥ 0.05.

H&E staining

FFPE samples were cut into 4-μm tissue sections and heated at 55°C prior to staining. Then, deparaffinization and rehydration was performed via incubation subsequently in xylene, 100%, 95%, 70%, 50%, and 25% ethanol. Then, slides were stained with hematoxylin for 6 minutes followed by washing with tap water for three times and bluing in Scott's Tap Water gently for 30 seconds. After that, tissues were counterstained with eosin for 30 seconds followed by washing with tap water. Then, the stained tissues were dried overnight at room temperature. The next day, sections were dehydrated with 100% ethanol and xylene. Finally, slides were sealed with nailpolish and images were taken using Leica DMi8 microscope .

Supplementary Data Fig. 1

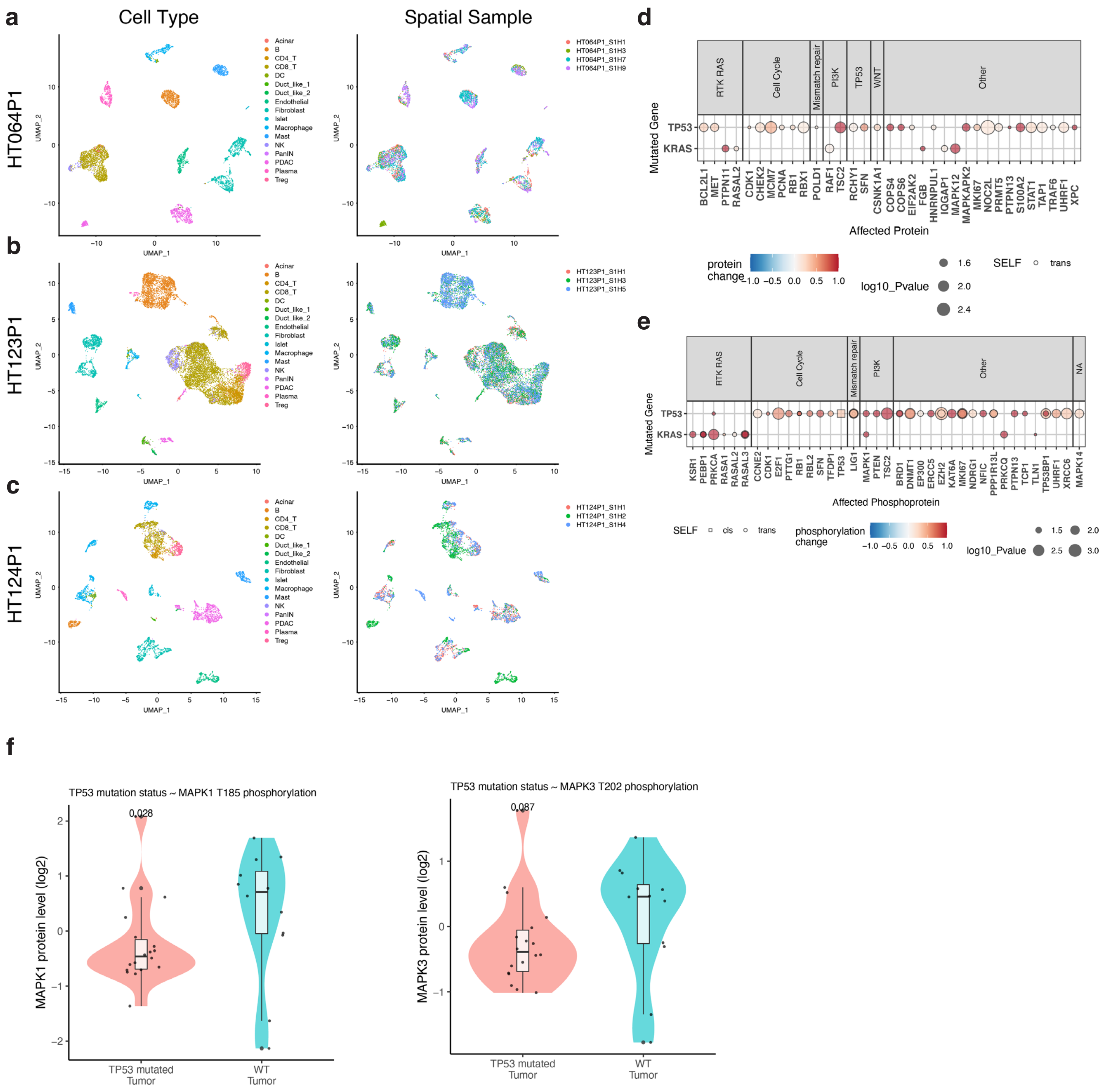

Supplementary Data Fig. 1. Proteogenomic Heterogeneity. **a)** Cell type (left) and spatial sample (right) UMAP distributions for case HT064P1. **b)** Same as panel A for case HT123P1. **c)** Same as panel A for case HT124P1. **d)** *KRAS* and *TP53* trans mutation impacts on protein levels. **e)** Same as panel D but for phosphosite levels. Overlapping dots denote several phosphosites from the same phosphoprotein. **f)** Phosphorylation levels of MAPK1 T185 and MAPK3 T202 sites between TP53 mutated and wild type tumor samples (n = 20 mutated samples, n = 10 wild type samples). The boxplots show the median with 1.5x IQR whiskers.

Supplementary Table 1: Clinical and demographic information for the cohort.

Supplementary Table 2: Cohort information including key clinical and molecular phenotypes.

Supplementary Table 3: Distances between spatial samples.

Supplementary Table 4: Details about proteomic TMT data.

Supplementary Table 5: Pathology annotation for all tumor pieces.

Supplementary Data 1: Bulk omics data including somatic and germline variants and proteogenomics data.

Supplementary Data 2: CNVkit raw copy number calls across the sample set.

Supplementary Data 3: Total cell count of transitional cell populations and mutation mapping across samples.

Supplementary Data 4: Differentially expressed genes (DEGs) identified by annotating spatial transcriptomics spots using the Loupe Browser and Seurat. FindAllMarkers function from Seurat was used to identify DEGs.

Supplementary Data 5: Tumor purity estimates predicted by ABSOLUTE across sample cohort.

References

1. Chatterjee, M. *et al.* Caveolin-1 is Associated with Tumor Progression and Confers a Multi-Modality Resistance Phenotype in Pancreatic Cancer. *Sci. Rep.* **5**, 10867 (2015).
2. Chen, D. & Che, G. Value of caveolin-1 in cancer progression and prognosis: Emphasis on cancer-associated fibroblasts, human cancer cells and mechanism of caveolin-1 expression (Review). *Oncol. Lett.* **8**, 1409–1421 (2014).
3. Liekens, S., Schols, D. & Hatse, S. CXCL12-CXCR4 axis in angiogenesis, metastasis and stem cell mobilization. *Curr. Pharm. Des.* **16**, 3903–3920 (2010).
4. Peitzsch, C., Cojoc, M., Kurth, I. & Dubrovska, A. Implications of CXCR4/CXCL12 Interaction for Cancer Stem Cell Maintenance and Cancer Progression. *Cancer Stem Cells: Emerging Concepts and Future Perspectives in Translational Oncology* 89–130 (2015) doi:10.1007/978-3-319-21030-8_4.
5. Eckert, F. *et al.* Potential Role of CXCR4 Targeting in the Context of Radiotherapy and Immunotherapy of Cancer. *Front. Immunol.* **9**, 3018 (2018).
6. Semenza, G. L. Targeting HIF-1 for cancer therapy. *Nature Reviews Cancer* vol. 3 721–732 (2003).
7. Huang, Y., Lin, D. & Taniguchi, C. M. Hypoxia inducible factor (HIF) in the tumor microenvironment: friend or foe? *Sci. China Life Sci.* **60**, 1114–1124 (2017).
8. Peng, J. *et al.* Single-cell RNA-seq highlights intra-tumoral heterogeneity and malignant progression in pancreatic ductal adenocarcinoma. *Cell Res.* **29**, 725–738 (2019).
9. Clark, D. J. *et al.* Integrated Proteogenomic Characterization of Clear Cell Renal Cell Carcinoma. *Cell* **180**, 207 (2020).
10. Mertins, P. *et al.* Reproducible workflow for multiplexed deep-scale proteome and phosphoproteome analysis of tumor tissues by liquid chromatography-mass spectrometry. *Nat. Protoc.* **13**, 1632–1661 (2018).

11. Griffith, M. *et al.* CIViC is a community knowledgebase for expert crowdsourcing the clinical interpretation of variants in cancer. *Nat. Genet.* **49**, 170–174 (2017).
12. Talevich, E., Shain, A. H., Botton, T. & Bastian, B. C. CNVkit: Genome-Wide Copy Number Detection and Visualization from Targeted DNA Sequencing. *PLoS Comput. Biol.* **12**, e1004873 (2016).
13. Carter, S. L. *et al.* Absolute quantification of somatic DNA alterations in human cancer. *Nat. Biotechnol.* **30**, 413–421 (2012).
14. Yoshihara, K. *et al.* Inferring tumour purity and stromal and immune cell admixture from expression data. *Nat. Commun.* **4**, 2612 (2013).
15. Leek, J. T., Johnson, W. E., Parker, H. S., Jaffe, A. E. & Storey, J. D. The sva package for removing batch effects and other unwanted variation in high-throughput experiments. *Bioinformatics* **28**, 882–883 (2012).
16. Aran, D., Hu, Z. & Butte, A. J. xCell: digitally portraying the tissue cellular heterogeneity landscape. *Genome Biol.* **18**, 220 (2017).
17. Dou, Y. *et al.* Proteogenomic Characterization of Endometrial Carcinoma. *Cell* **180**, 729–748.e26 (2020).
18. Bailey, M. H. *et al.* Comprehensive Characterization of Cancer Driver Genes and Mutations. *Cell* **174**, 1034–1035 (2018).
19. Sanchez-Vega, F. *et al.* Oncogenic Signaling Pathways in The Cancer Genome Atlas. *Cell* **173**, 321–337.e10 (2018).
20. Eser, S., Schnieke, A., Schneider, G. & Saur, D. Oncogenic KRAS signalling in pancreatic cancer. *British Journal of Cancer* vol. 111 817–822 (2014).
21. Collisson, E. A. *et al.* A central role for RAF→MEK→ERK signaling in the genesis of pancreatic ductal adenocarcinoma. *Cancer Discov.* **2**, 685–693 (2012).
