# Supplementary material for: Spatially restricted drivers and transitional cell populations cooperate with the microenvironment in untreated and chemo-resistant pancreatic cancer
Source: Nat Genet. 2022 Aug 22;54(9):1390–405. doi: 10.1038/s41588-022-01157-1 (PMC9470535; doi:10.1038/s41588-022-01157-1)
Supplement: Supplementary file 2 — Reporting Summary [file 41588_2022_1157_MOESM2_ESM.pdf]

nature portfolio

Corresponding author(s): Li Ding, Ryan C. Fields, David G. DeNardo

Last updated by author(s): May 4, 2022

Reporting Summary

Nature Portfolio wishes to improve the reproducibility of the work that we publish. This form provides structure for consistency and transparency in reporting. For further information on Nature Portfolio policies, see our Editorial Policies and the Editorial Policy Checklist.

Statistics

For all statistical analyses, confirm that the following items are present in the figure legend, table legend, main text, or Methods section.

n/a	Confirmed	
☐	☒	The exact sample size (*n*) for each experimental group/condition, given as a discrete number and unit of measurement
☐	☒	A statement on whether measurements were taken from distinct samples or whether the same sample was measured repeatedly
☐	☒	The statistical test(s) used AND whether they are one- or two-sided<br>*Only common tests should be described solely by name; describe more complex techniques in the Methods section.*
☐	☒	A description of all covariates tested
☐	☒	A description of any assumptions or corrections, such as tests of normality and adjustment for multiple comparisons
☐	☒	A full description of the statistical parameters including central tendency (e.g. means) or other basic estimates (e.g. regression coefficient) AND variation (e.g. standard deviation) or associated estimates of uncertainty (e.g. confidence intervals)
☐	☒	For null hypothesis testing, the test statistic (e.g. *F*, *t*, *r*) with confidence intervals, effect sizes, degrees of freedom and *P* value noted<br>*Give P values as exact values whenever suitable.*
☒	☐	For Bayesian analysis, information on the choice of priors and Markov chain Monte Carlo settings
☒	☐	For hierarchical and complex designs, identification of the appropriate level for tests and full reporting of outcomes
☐	☒	Estimates of effect sizes (e.g. Cohen's *d*, Pearson's *r*), indicating how they were calculated

Our web collection on statistics for biologists contains articles on many of the points above.

Software and code

Policy information about availability of computer code

Data collection	No software was used for data collection.
Data analysis	ABSOLUTE v1.0.6, (Carter et al., 2012), https://software.broadinstitute.org/cancer/cga/absolute<br>Ascore v1.0.6858, (Beausoleil et al., 2006), https://github.com/PNNL-Comp-Mass-Spec/AScore<br>bam-readcount v0.8, McDonnell Genome Institute, https://github.com/genome/bam-readcount<br>Bioconda, (The Bioconda Team et al., 2018), https://bioconda.github.io/<br>Bioconductor v3.9, (Huber et al., 2015), https://bioconductor.org/<br>CellPhoneDB, (Efremova et al., 2020), https://www.cellphonedb.org<br>Cell Ranger v3.1.0, 10X Genomics, https://support.10xgenomics.com/single-cell-gene-expression/software/pipelines/latest/what-is-cell-ranger<br>Space Ranger v1.1.0, 10X Genomics, https://support.10xgenomics.com/spatial-gene-expression/software/pipelines/latest/what-is-space-ranger<br>CharGer v0.5.4, (Scott et al., 2019), https://github.com/ding-lab/CharGer<br>clusterProfiler v3.8.1, (Yu et al., 2012), https://bioconductor.org/packages/release/bioc/html/clusterProfiler.html<br>ConsensusClusterPlus v1.56.0, (Wilkerson and Hayes, 2010), https://bioconductor.org/packages/release/bioc/html/ConsensusClusterPlus.html<br>data.table_1.12.6 , R Development Core Team, https://cran.r-project.org/package=data.table<br>dendsort_0.3.3, (Sakai et al., 2014), https://cran.r-project.org/package=dendsort<br>dplyr_0.8.5, R Development Core Team, https://cran.r-project.org/package=dplyr<br>ESTIMATE, (Yoshihara et al., 2013), https://sourceforge.net/projects/estimateproject/<br>GATK v4.0.0.0, (McKenna et al., 2010), https://github.com/broadgsa/gatk<br>germlinewrapper v1.1, Li Ding Lab, https://github.com/ding-lab/germlinewrapper<br>ggplot2_3.3.2, R Development Core Team, https://CRAN.R-project.org/package=ggplot2<br>gridExtra_2.3, R Development Core Team, https://cran.r-project.org/package=gridExtra<br>bulk-RNA-Seq expression, Li Ding Lab, https://github.com/ding-lab/HTAN_bulkRNA_expression

March 2021

inferCNV v0.8.2, (Tickle et al., 2019), https://github.com/broadinstitute/infercnv
Integrative Genomics Viewer, (Robinson et al., 2011), https://igv.org
Loupe Browser v.5.0, 10X genomics, https://www.10xgenomics.com/products/loupe-browser
magrittr_1.5, R Development Core Team, https://cran.r-project.org/package=magrittr
Matrix_1.2-17 , R Development Core Team, https://CRAN.R-project.org/package=Matrix
MEDALT, (Wang et al., 2020), https://github.com/KChen-lab/MEDALT
MuTect v1.1.7, (Cibulskis et al., 2013), https://github.com/broadinstitute/mutect
pheatmap_1.0.12, R Development Core Team, https://cran.r-project.org/package=pheatmap
Pindel v0.2.5, (Ye et al., 2009), https://github.com/genome/pindel
Python v3.7, Python Software Foundation, https://www.python.org/
R v3.6, R Development Core Team, https://www.r-project.org/
RColorBrewer_1.1-2, R Development Core Team, https://CRAN.R-project.org/package=RColorBrewer
RCTD v.1.2.0, (Cable et al., 2021), https://github.com/dmcable/RCTD
reshape2_1.4.3, R Development Core Team, https://cran.r-project.org/package=reshape2
Samtools v1.2, (Li et al., 2009), https://www.htslib.org/
scVarScan, Li Ding Lab, https://github.com/ding-lab/10Xmapping
SeqQEst, Li Ding Lab, https://github.com/ding-lab/SeqQEst
Seurat v3.1.2 and v4.0.3, (Butler et al., 2018), https://cran.r-project.org/web/packages/Seurat
somaticwrapper v1.5, Li Ding Lab, https://github.com/ding-lab/somaticwrapper
sva v3.40.0, (Huber et al., 2015), https://bioconductor.org/packages/release/bioc/html/sva.html
STAR v2.7.4a, (Dobin et al., 2013), https://github.com/alexdobin/STAR
Strelka v2.9.2, (Kim et al., 2018), https://github.com/Illumina/strelka
stringr_1.4.0, R Development Core Team, https://cran.r-project.org/package=stringr
Subread v2.0.1, (Liao et al., 2013), https://sourceforge.net/projects/subread/
Tidyverse, (Wickham et al., 2019), https://www.tidyverse.org/
VarScan v2.3.8, (Koboldt et al., 2012), https://dkoboldt.github.io/varscan/
viridis_0.5.1, R Development Core Team, https://github.com/sjmgarnier/viridis
viridisLite_0.3.0, R Development Core Team, https://github.com/sjmgarnier/viridis
xCell v1.2, (Aran et al., 2017), http://xCell.ucsf.edu/
Monocle3 v3.10, https://cole-trapnell-lab.github.io/monocle3/docs/installation/
CopyKat 1.0.4 (Gao et al., 2021) ,https://github.com/navinlabcode/copykat
inferCNV post processing, https://github.com/ding-lab/infer_cnv_postprocesssing.git

For manuscripts utilizing custom algorithms or software that are central to the research but not yet described in published literature, software must be made available to editors and reviewers. We strongly encourage code deposition in a community repository (e.g. GitHub). See the Nature Portfolio guidelines for submitting code & software for further information.

Data

Policy information about availability of data

All manuscripts must include a data availability statement. This statement should provide the following information, where applicable:

- Accession codes, unique identifiers, or web links for publicly available datasets
- A description of any restrictions on data availability
- For clinical datasets or third party data, please ensure that the statement adheres to our policy

All raw image and sequencing data will be deposited into the publicly available HTAN DCC at https://data.humantumoratlas.org/. References (GRCh38 genome reference v3.0.0 and refdata-gex-mm10-2020-A) used for single-cell for the human and mouse genomes, respectively are available https://support.10xgenomics.com/single-cell-gene-expression/software/release-notes/build. Mouse single-cell RNA-seq data is available using the following BioProject PRJNA835747. Data for single-cell integration from Peng et al. was downloaded from the Genome Sequence Archive under project PRJCA001063.

Field-specific reporting

Please select the one below that is the best fit for your research. If you are not sure, read the appropriate sections before making your selection.

☒ Life sciences ☐ Behavioural & social sciences ☐ Ecological, evolutionary & environmental sciences

For a reference copy of the document with all sections, see nature.com/documents/nr-reporting-summary-flat.pdf

Life sciences study design

All studies must disclose on these points even when the disclosure is negative.

Sample size

Tumor samples were collected as patients underwent surgery during the collection time period on a rolling basis. The sample size in the manuscript is comparable or greater than most studies published to date. Tumor samples were collected as patients underwent surgery during the collection time period on a rolling basis, and thus no sample size was calculated. As the goal was to build an atlas, rather than to obtain sufficient statistical power, we included as many samples as we could up until manuscript submission. Nevertheless, our sample sizes ($n > 80$ patients; $n > 230$k cells) are comparable or much greater than most single cell studies published to date.

Data exclusions

We excluded two bulk RNA-Seq samples that did not pass quality control (QC) based on FASTQC and HLA-typing checks.

Replication

We analyzed previously published data on single cell pancreatic cancer studies to ensure that our cell typing is consistent. We included a cohort of 10 tumors that underwent single nucleus RNA sequencing and used these results to replicate/validate our single cell findings.

	Additionally, we validated results using orthogonal technologies such as immunofluoresence staining and spatial transcriptomics. Validated results included cell type assignment, differentially expressed genes in cell types of interest, and staining for the presence of key markers in sample-matched tissue specimens.
Randomization	Due to our patient accrual on a rolling basis, we did not 'allocate' patients into specific treatment groups, thus the imbalance in the number of patients in each treatment group (e.g. we have a high number of samples in the FOLFIRINOX-treated group). Moreover, the study was designed around discovery and characterization, rather than clinical comparison of treatment groups, e.g. in terms of survival. Consequently, there was no issue of controlling for covariates to reduce statistical noise for improving regression. Sequencing was done in randomized batches and we corrected proteomic batch effects (due to TMT plexes) using ComBat. Mice from our KPC-OG breeding colony were genotyped for all alleles. Three mice with the with full genotype (p48-Cre+;LSL-KRASG12D+;p53flox;OVA-GFP+) were allocated into the KPC-OG tumor group. p48-Cre(-) littermate controls were used for non-cancer control groups, and randomly allocated into either the normal pancreas (no treatment) or pancreatitis (cerulein treated) group.
Blinding	Blinding was not relevant in our study as samples were collected from patients as they came into the clinic. As every sample was included in the study and processed in the same standardized way, knowledge of clinical phenotypes would not affect the study.

Reporting for specific materials, systems and methods

We require information from authors about some types of materials, experimental systems and methods used in many studies. Here, indicate whether each material, system or method listed is relevant to your study. If you are not sure if a list item applies to your research, read the appropriate section before selecting a response.

Materials & experimental systems

n/a	Involved in the study	
☐	☒	Antibodies
☒	☐	Eukaryotic cell lines
☒	☐	Palaeontology and archaeology
☐	☒	Animals and other organisms
☐	☒	Human research participants
☒	☐	Clinical data
☒	☐	Dual use research of concern

Methods

n/a	Involved in the study	
☒	☐	ChIP-seq
☒	☐	Flow cytometry
☒	☐	MRI-based neuroimaging

Antibodies

Antibodies used	α-Amylase, Sigma, Catalog: A8273<br>Cytokeratin 19, Santa Cruz, Catalog: sc-376126<br>Ki67, Thermo, Catalog: 14-5698-82<br>Alexa Fluor 488 AffiniPure F(ab')2 Fragment DαRabbit, Jackson Immuno Research, Catalog: 711-546-152<br>Alexa Fluor 555 AffiniPure F(ab')2 Fragment DαMouse, Thermo, Catalog: A31570<br>Alexa Fluor 647 AffiniPure F(ab')2 Fragment DαRat, Jackson Immuno Research, Catalog: 712-606-153<br>Hoechst, Life Technologies, Catalog: H3570
Validation	α-Amylase, manufactured by Sigma (A8273) - validation data in the following PMID: 34358441<br>Cytokeratin 19, manufactured by Santa Cruz - validation data in the following PMID: 33951479<br>Ki67, manufactured by Thermo - validation data in the following PMID: 32750316

Animals and other organisms

Policy information about studies involving animals; ARRIVE guidelines recommended for reporting animal research

Laboratory animals	The breed of mice are C57BL/6, all male and pancreatic tissue was extracted at 4 months.
Wild animals	N/A
Field-collected samples	N/A
Ethics oversight	Washington University School of Medicine IACUC under protocol #19-0856

Note that full information on the approval of the study protocol must also be provided in the manuscript.

Human research participants

Policy information about studies involving human research participants

Population characteristics	All patients involved in this study were between the ages of 38-82 with pancreatic ductal adenocarcinoma. Patients were either treatment naive or had neoadjuvant therapy prior to sample collection. Detailed clinical information can be found in Supplementary Table 1.

Recruitment	Participants for this study were recruited from all patients undergoing surgical resection of primary pancreatic ductal adenocarcinoma, and were screened only on the basis of resectable disease identified by the schedule contained within the electronic health record system (EPIC). Clinical research staff conducted a manual review to ensure each participant was assigned a number and consented either through the Hepato-Biliary clinic at the Washington University in St. Louis, Department of Surgery and Barnes Jewish Hospital or on the day of surgery. All patients provided informed consent approved by the Washington University in St. Louis Institutional Review Board.
Ethics oversight	Primary pancreatic adenocarcinomas either treatment naïve or neoadjuvant treated (Supplementary Table S1) were collected with written consent from all patients under the protocol 11-08117 with approval from the Washington University in St. Louis Institutional Review Board. Consent included the use of all de-identified patient data for publication. Participants were not compensated.

Note that full information on the approval of the study protocol must also be provided in the manuscript.
